# Nicotinic Acetylcholine Receptors Control Encoding and Retrieval of Associative Recognition Memory through Plasticity in the Medial Prefrontal Cortex

**DOI:** 10.1016/j.celrep.2018.03.016

**Published:** 2018-03-27

**Authors:** Marie H. Sabec, Susan Wonnacott, E. Clea Warburton, Zafar I. Bashir

**Affiliations:** 1 School of Physiology, Pharmacology and Neuroscience, University of Bristol, Bristol BS8 1TD, UK; 2 Department of Biology and Biochemistry, University of Bath, Bath BA2 7AY, UK

**Keywords:** acetylcholine, nicotinic receptor, medial prefrontal cortex, associative recognition memory, plasticity

## Abstract

Nicotinic acetylcholine receptors (nAChRs) expressed in the medial prefrontal cortex have critical roles in cognitive function. However, whether nAChRs are required for associative recognition memory and the mechanisms by which nAChRs may contribute to mnemonic processing are not known. We demonstrate that nAChRs in the prefrontal cortex exhibit subtype-specific roles in associative memory encoding and retrieval. We present evidence that these separate roles of nAChRs may rely on bidirectional modulation of plasticity at synaptic inputs to the prefrontal cortex that are essential for associative recognition memory.

## Highlights

• Prefrontal α7 nAChRs are critical for encoding of associative recognition memory
• Prefrontal α4β2 nAChRs are required for retrieval of associative recognition memory
• α7 and α4β2 nAChRs gate bidirectional plasticity at hippocampal-prefrontal synapses
• Bidirectional plasticity underlies the role of nAChR in associative recognition

## Graphical Abstract

**Figure:**
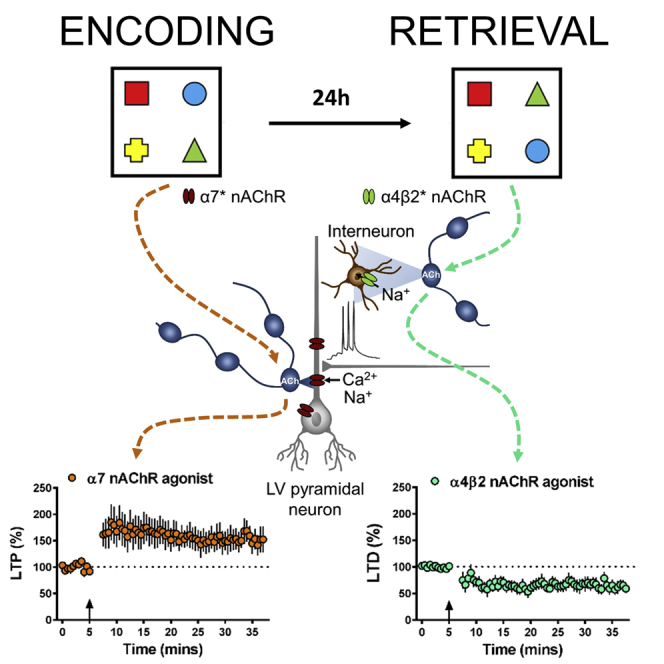

## Teaser

Sabec et al. reveal a divergence in function of prefrontal nicotinic receptor subtypes in different stages of long-term associative recognition memory that relates to bidirectional modulation of synaptic plasticity at hippocampal-prefrontal synapses.

## Introduction

Associative visual recognition is the ability to integrate the identity of an object with the location in which it was encountered (Dickerson and Eichenbaum, 2010). Associative recognition memory consists of initial encoding and subsequent retrieval and depends on the medial prefrontal cortex (mPFC) integrating information received from the hippocampus (HPC) and other brain regions (Barker et al., 2007, Barker et al., 2017).

Acetylcholine is essential for a variety of complex behaviors such as the performance of attention and learning tasks (Wallace and Bertrand, 2013, Parikh et al., 2007), and cholinergic deficits are central to the etiology of dementias (Picciotto and Zoli, 2002). To date, there has been a focus on muscarinic acetylcholine receptors in mPFC-dependent memory (Barker and Warburton, 2008). However, it is not known whether nicotinic acetylcholine receptors (nAChRs) in the mPFC play any role in encoding, consolidation, or retrieval of associative recognition memory in rats.

Synaptic plasticity is considered essential for learning and memory (Martin et al., 2000). nAChRs are expressed throughout the mPFC (Poorthuis et al., 2013), and their activation can give rise to synaptic plasticity (Verhoog et al., 2016, Couey et al., 2007, Udakis et al., 2016). HPC input to the mPFC is crucial for associative recognition memory (Barker et al., 2017), but whether activation of nAChRs governs synaptic plasticity at HPC-mPFC synapses and how such nicotinic modulation may be involved in distinct phases of associative recognition memory are not known.

We now test the hypothesis that specific nAChRs induce different forms of synaptic plasticity to bring about encoding and retrieval of associative recognition memory. We report that homomeric α7 nAChRs are essential for both encoding of associative recognition and induction of long-term potentiation (LTP) of HPC-mPFC synapses. In contrast, α4β2-containing (α4β2^∗^) nAChRs are essential for both retrieval of associative memory and long-term depression (LTD). Selective inhibition of LTP or LTD expression mechanisms prevented memory encoding and retrieval, respectively. We conclude that different nAChRs in the mPFC promote LTP or LTD of HPC-mPFC synapses to enable encoding or retrieval of associative recognition memory.

## Results

### α7 nAChRs Are Required for Encoding and α4β2 nAChRs for Retrieval of Associative Recognition Memory

**Figure 1:**
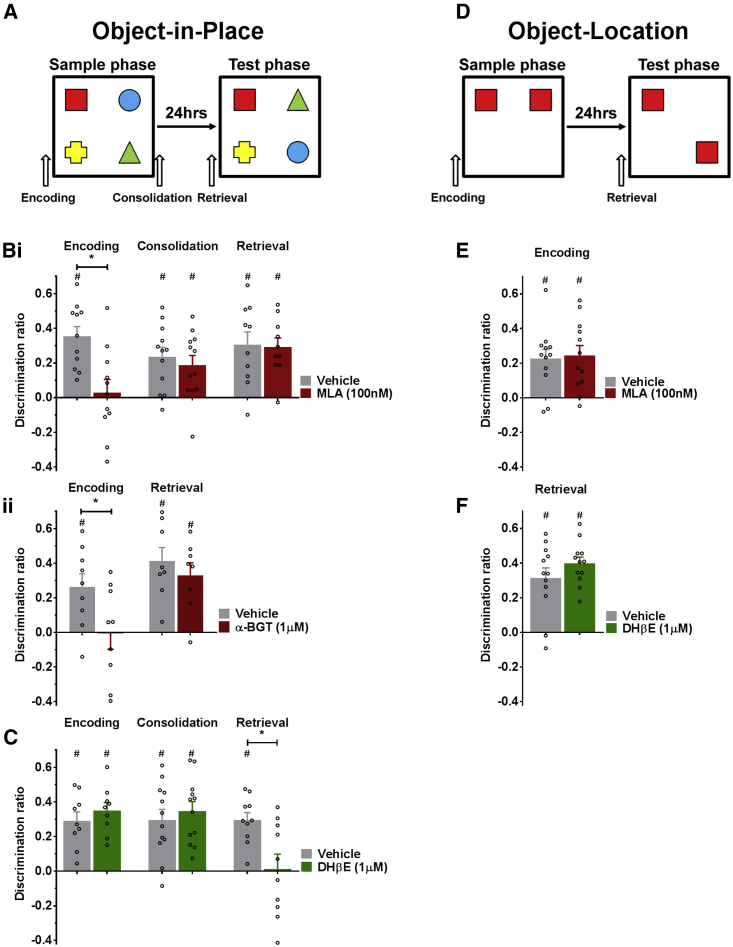
Associative Recognition Memory Is Dependent on Activation of Different nAChRs in the mPFC (A) Schematic of OiP task. Arrows represent the timing of mPFC infusions. (B) MLA impaired OiP memory when infused before the sample phase (encoding; n = 11), but not after the sample phase (consolidation; n = 12) or before the test phase (retrieval; n = 10) (i). α-BGT also impaired discrimination when infused before the sample phase (n = 9), but not before the test phase (n = 8) (ii). (C) DHβE impaired OiP memory if given before the test phase (n = 10), but not before (n = 9) or after (n = 12) the sample phase. (D) Schematic of the OL task (E and F) OL memory was not impaired by MLA infusion before the sample phase (n = 12) (E) or DHβE infusion before the test phase (n = 12) (F). Data are presented as mean ± SEM (^∗^p < 0.05 paired t test; #p < 0.05 one-sample t test against 0). See also Tables S1 and S2.

Selective antagonists of α7 and α4β2^∗^ receptors were infused intra-cortically into mPFC during different phases of the object-in-place (OiP) task (Figure 1A). The α7 nAChR antagonist methyllycaconitine citrate (MLA) (100 nM), when given prior to the sample phase, impaired OiP discrimination (MLA versus vehicle [Veh] t_(10)_ = 2.756, p = 0.021). Thus, following MLA, discrimination was not different from chance, while the vehicle group had a significant discrimination (MLA t_(10)_ = 0.372, p = 0.717; Veh t_(10)_ = 6.368, p < 0.001; Figure 1Bi). In contrast, there were no deficits in OiP when MLA or vehicle was delivered after the sample phase (MLA t_(11)_ = 3.335, p = 0.007; Veh t_(11)_ = 4.382, p = 0.001; MLA versus Veh t_(11)_ = 0.820, p = 0.429; Figure 1Bi) or prior to the test phase (MLA t_(9)_ = 5.559, p < 0.001; Veh t_(9)_ = 4.145, p = 0.003; MLA versus Veh t_(9)_ = 0.190, p = 0.854; Figure 1Bi).

To confirm these effects, the experiment was repeated with α-bungarotoxin (α-BGT; 1 μM). Infusion of α-BGT prior to the sample phase impaired OiP encoding (α-BGT versus Veh t_(8)_ = 2.559, p = 0.034), and animals failed to discriminate (α-BGT t_(8)_ = −0.075, p = 0.942; Veh t(8) = 3.419, p = 0.009; Figure 1Bii). There was no deficit in OiP when α-BGT or vehicle was delivered prior to the test phase (α-BGT t_(7)_ = 4.601, p = 0.002; Veh; t_(7)_ = 5.360, p = 0.001; α-BGT versus Veh t_(7)_ = 1.276, p = 0.243; Figure 1Bii). Thus, α7 nAChRs in mPFC are critical for encoding, but not for consolidation or retrieval of long-term associative recognition memory.

Infusion of the α4β2^∗^ nAChR antagonist DHβE (1 μM) impaired discrimination when given prior to the test phase (DHβE t_(9)_ = 0.141, p = 0.891; Veh t_(9)_ = 6.954, p < 0.001; Figure 1C); there was a significant difference between DHβE and vehicle (DHβE versus Veh t_(9)_ = 2.467, p = 0.036). Memory was not impaired following administration of DHβE either prior to the sample phase (DHβE t_(8)_ = 7.643, p < 0.001; Veh t_(8)_ = 5.593, p = 0.001; DHβE versus Veh t_(8)_ = −1.085, p = 0.310) or after the sample phase (DHβE t_(11)_ = 6.342, p < 0.001; Veh t_(11)_ = 4.831, p = 0.001; DHβE versus Veh t_(11)_ = 0.-606, p = 0.557; Figure 1C). Thus, α4β2^∗^ nAChRs in the mPFC are critical for the retrieval of long-term associative recognition memory, but not for its consolidation or encoding.

There was no difference in total object exploration during sample or test phases during either α7 nAChR or α4β2^∗^ nAChR antagonism (Table S1), indicating the drugs had no effect on motor function or exploratory behavior.

To ensure effects of nAChR inhibition were not due to deficits in attention (Wallace and Bertrand, 2013), animals were tested on a non-associative object location (OL) task (Figure 1D) that is independent of the mPFC (Barker et al., 2007). Infusion of MLA prior to sample phase or DHβE prior to test phase had no effect on OL memory (Figures 1E and 1F; MLA t_(11)_ = 4.220, p = 0.001; Veh t_(11)_ = 4.263, p = 0.001; MLA versus Veh t_(11)_ = −0.236, p = 0.818; DHβE t_(11)_ = 11.193, p < 0.001; Veh t_(11)_ = 5.366, p < 0.001; DHβE versus Veh t_(11)_ = −1.188, p = 0.260). In addition, neither MLA nor DHβE had any effect on total exploration times (Table S2).

### α7 and α4β2 nAChRs Are Required for LTP and LTD, Respectively

**Figure 2:**
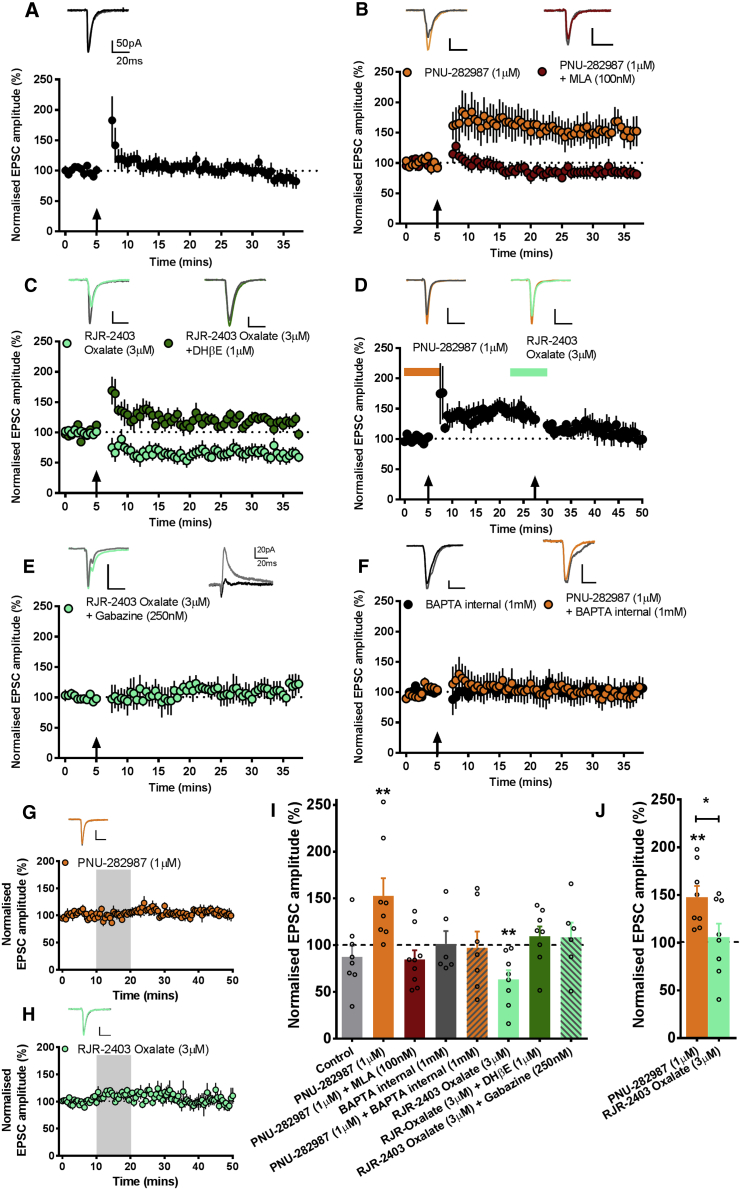
nAChRs Bidirectionally Modulate HPC-mPFC Plasticity (A) Transient potentiation following delivery of STDP, indicated by arrow (n = 8). (B) LTP induced by combined STDP and PNU-282987 (n = 8) was blocked in separate experiments by co-application of MLA (n = 9). (C) LTD induced by STDP with RJR-2403 oxalate (n = 8) was blocked by co-application of DHβE (n = 8). (D) Induction of PNU-282987 STDP LTP was reversed by RJR-2403 Oxalate STDP induced LTD (n = 8). (E and F) α4β2 nAChR LTD was blocked in the presence of gabazine (n = 6) (E), and α7 nAChR LTP was blocked by postsynaptic BAPTA (n = 7) (F). (G and H) Application of PNU-282987 (n = 6) (G) or RJR-2403 oxalate (n = 6) (H) in the absence of STDP did not induce plasticity. In all graphs, representative EPSCs are shown from baseline (gray traces) and the last 5 min (colored traces) of the experiment. (I and J) Summary of normalized EPSC amplitudes recorded in the final 5 min of each STDP experiment. Data are presented as normalized mean ± SEM (^∗^p < 0.05, ^∗∗^p < 0.01; paired t test in I or repeated-measures ANOVA with Bonferroni post hoc in J).

To probe how nAChRs may contribute to separate phases of associative recognition we examined, *in vitro*, synaptic plasticity at the HPC-mPFC pathway (Banks et al., 2015) that is essential for OiP memory (Barker et al., 2017). A spike-timing-dependent plasticity protocol (STDP; Parent et al., 2010) resulted only in a transient increase in HPC-mPFC EPSCs (t_(7)_ = −0.410, p = 0.694; Figures 2A and 2I). To test whether nAChR subtypes can regulate synaptic plasticity, selective agonists were applied with STDP. In the presence of the α7 nAChR agonist PNU-282987 (1 μM) LTP was induced by STDP (t_(7)_ = 4.059, p = 0.005; Figures 2B and 2I). LTP was prevented by co-application of α7 nAChR antagonist MLA (100 nM) (t_(8)_ = −1.583, p = 0.152; Figures 2B and 2I) or intracellular 1,2-bis(o-aminophenoxy)ethane-N,N,N′,N′-tetraacetic acid (BAPTA) (1 mM) (t_(6)_ = −0.237, p = 0.821; control t_(5)_ = −0.708, p = 0.510; Figures 2F and 2I).

LTD was induced when STDP was delivered in the presence of the α4β2^∗^ agonist RJR-2403 oxalate (3 μM) (t_(7)_ = −4.518, p = 0.003; Figures 2C and 2I); LTD was prevented by co-application of the α4β2^∗^ antagonist DHβE (1 μM) (t_(7)_ = 1.100, p = 0.308; Figures 2C and 2I). Interestingly, α4β2^∗^ nAChR activation coupled with STDP reversed prior α7 nAChR induced LTP (F_(2,14)_ = 8.963, p = 0.003; Figures 2D and 2J). Moreover, LTD was blocked in the presence of gabazine (250 nM) (t_(5)_ = 0.651, p = 0.544; Figures 2E and 2I), suggesting GABAergic transmission is required for LTD induction.

Neither α7 nor α4β2^∗^ nAChR agonists affected synaptic transmission in the absence of STDP (PNU t_(5)_ = −0.489, p = 0.645; RJR t_(5)_ = −1.126, p = 0.311; Figures 2G and 2H). Together, these results demonstrate that paired pre- and postsynaptic activity combined with α7 or α4β2^∗^ nAChR activation differentially induces LTP and LTD at the HPC-mPFC pathway. LTP may rely on α7 nAChR-mediated increases in intracellular calcium and LTD on α4β2^∗^ nAChR-mediated GABAergic inhibition.

### Expression of α7 nAChR LTP Is Dependent on Atypical PKCs, while α4β2 nAChR LTD Requires GluA2 Internalization

**Figure 3:**
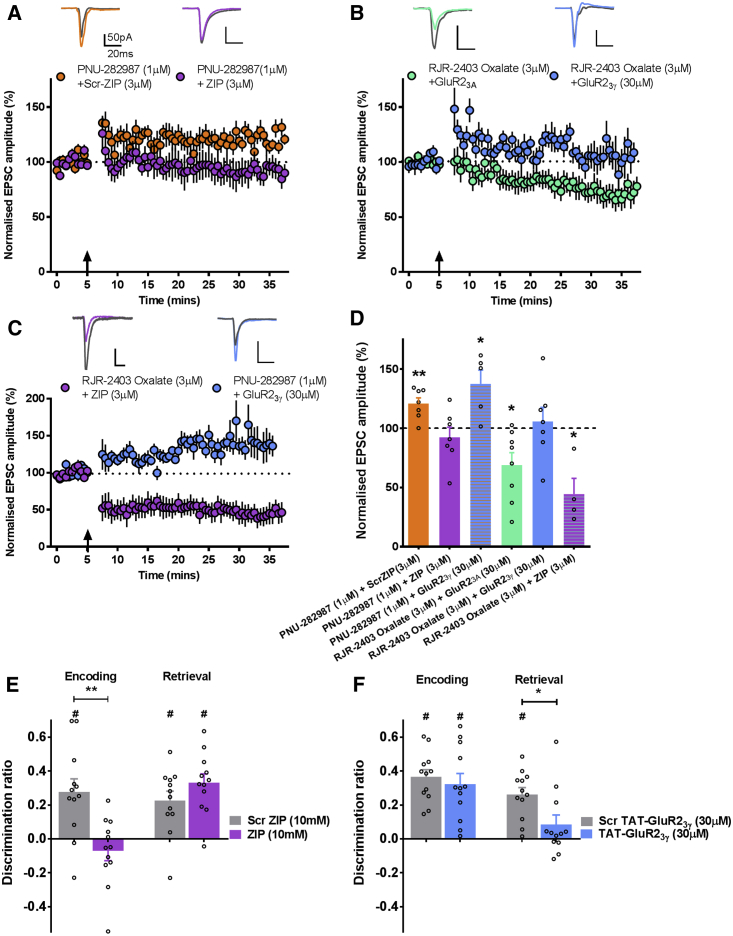
Disrupting Expression of LTP or LTD Blocks α7-Induced LTP and α4β2-Induced LTD and Blocks Associative Memory Encoding and Retrieval, Respectively (A) ZIP (n = 7), but not Scr-ZIP (n = 7), prevented STDP/PNU-282987 induction of LTP. (B) GluR2_3γ_ (n = 7), but not GluR2_3A_ (n = 8), prevented STDP/DHβE induction of LTD. (C) α7 nAChR LTP was not blocked by GluR2_3γ_ (n = 5), and α4β2 nAChR LTD was not blocked by ZIP (n = 4). Representative traces shown from baseline and the last 5 min of the experiment. (D) Summary of normalized EPSC amplitudes recorded in the final 5 min of each experiment. (E) ZIP impaired OiP memory when infused before the sample phase (n = 12), but not the test phase (n = 12). (F) TAT-GluR2_3__γ_ impaired OiP when infused before the test phase (n = 12), but not the sample phase (n = 12). The average discrimination following two trials with TAT-GluR2_3__γ_/Scr TAT-GluR2_3__γ_ infusion before the test phase is shown. Data are presented as mean ± SEM (^∗^p < 0.05, ^∗∗^p < 0.01 paired t test; #p < 0.05 one-sample t test against 0). See also Table S3.

To test whether bidirectional plasticity may provide a mechanism by which different nAChR subtypes contribute to associative recognition, we first determined whether α7 and α4β2^∗^ nAChR-gated plasticity could be blocked by selective manipulation of intracellular mechanisms that mediate LTP or LTD expression. Zeta inhibitory peptide (ZIP) blocks LTP through inhibition of atypical PKC isoforms PKCι/λ and PKMζ (Serrano et al., 2005, Ren et al., 2013). Loading mPFC pyramidal cells with ZIP (3 μM) via the recording electrode blocked α7 nAChR-dependent LTP (t_(6)_ = −0.975, p = 0.367; Figures 3A and 3D). In contrast, α7 nAChR-LTP was not blocked by postsynaptic loading of scrambled ZIP (t_(6)_ = 4.047, p = 0.007; Figures 3A and 3D)

To test expression mechanisms of α4β2^∗^ nAChR-induced LTD, we used the peptide GluR2_3γ_ (30 μM) to inhibit activity-driven endocytosis without affecting basal transmission or LTP (Ahmadian et al., 2004, Brebner et al., 2005). Postsynaptic loading of GluR2_3γ_ blocked α4β2^∗^ nAChR-dependent LTD (t_(6)_ = 0.470, p = 0.655; Figures 3B and 3D). LTD was not blocked by the inactive peptide, GluR2_3A_ (t_(7)_ = −3.215, p = 0.015; Figures 3B and 3D)

α7 nAChR-induced LTP was not affected by GluR2_3γ_ (30 μM) (t_(4)_ = 3.290, p = 0.030; Figures 3C and 3D), and α4β2^∗^ nAChR-dependent LTD was not affected by ZIP (3 μM) (t_(3)_ = −4.504, p = 0.020; Figures 3C and 3D), thus confirming the selectivity of the GluR2_3γ_ for LTD and ZIP for LTP.

### Blocking Expression Mechanisms of LTP and LTD Prevents Encoding and Retrieval of Associative Recognition Memory, Respectively

We next tested the hypothesis that if the different forms of nAChR-induced plasticity are essential for the separate phases of associative recognition memory, then selective blockade of LTP and LTD expression mechanisms *in vivo* should result in selective deficits in encoding and retrieval, respectively.

Infusion of ZIP (10 mM) prior to the sample phase impaired OiP performance compared to scrambled ZIP (Scr-ZIP; 10 mM) (ZIP versus Scr-ZIP t_(11)_ = 3.293, p = 0.004); discrimination following ZIP was not different from chance (ZIP t_(11)_ = −1.189, p = 0.260; Scr-ZIP t_(11)_ = 3.622, p = 0.004; Figure 3E). In contrast, memory was not impaired when ZIP was infused prior to the test phase (ZIP t_(11)_ = 6.491, p < 0.001; Scr-ZIP t_(11)_ = 4.095, p = 0.002; ZIP versus Scr-ZIP t_(11)_ = −1.553, p = 0.149; Figure 3E). Therefore, selective blockade of LTP in the mPFC causes a deficit in associative recognition memory encoding but is without effect on memory retrieval.

Delivery of TAT-GluR2_3γ_ (30 μM) (Cazakoff and Howland, 2011) or control peptide (Scr TAT-GluR2_3γ_; 30 μM) had no effect on OiP discrimination when delivered prior to the sample phase (TAT-GluR2_3γ_ t_(11)_ = 5.204, p < 0.001; Scr TAT-GluR2_3γ_ t_(11)_ = 8.670, p < 0.001; TAT-GluR2_3γ_ versus Scr TAT-GluR2_3γ_ t_(11)_ = 0.716, p = 0.489; Figure 3F). In contrast, infusions given before the test phase produced a significant difference in memory performance between conditions (TAT-GluR2_3γ_ versus Scr TAT-GluR2_3γ_ t_(11)_ = 2.251, p = 0.046). Thus performance under TAT-GluR2_3γ_ did not differ from chance (TAT-GluR2_3γ_ t_(11)_ = 1.527, p = 0.115) in contrast to control performance (Scr TAT-GluR2_3γ_ t_(11)_ = 6.423, p < 0.001; Figure 3F). Selective blockade of LTD in the mPFC therefore causes a deficit in long-term associative recognition memory retrieval but is without effect on memory encoding.

The deficits in memory resulting from blocking expression of plasticity were not a result of motor or attentional impairment, as total object exploration was equivalent between active and inactive peptide conditions for both ZIP and GluR2_3γ_ (Table S3). Therefore, blockade of LTP (dependent on α7 nAChR) and LTD (dependent on α4β2^∗^ nAChR) resulted in selective deficits in the encoding and retrieval, respectively, of OiP memory.

## Discussion

Our study takes advantage of the temporal specificity of pharmacological interventions to enable transient receptor inactivation and demonstrates that homomeric α7 and heteromeric α4β2^∗^ nAChR subtypes make differential contributions to cognitive functions and to underlying synaptic plasticity at the HPC-mPFC pathway. These results suggest that different nAChRs promote LTP or LTD to enable encoding or retrieval of associative recognition memory.

Within the mPFC, α7 and α4β2^∗^ subtypes of nAChRs display differential expression across distinct cells and layers (Couey et al., 2007, Poorthuis et al., 2013, Verhoog et al., 2016, Wallace and Bertrand, 2013). In the current work, we focus on HPC input to pyramidal cells in layer V of the mPFC, since this input is crucial for associative recognition memory (Barker et al., 2017).

**Figure 4:**
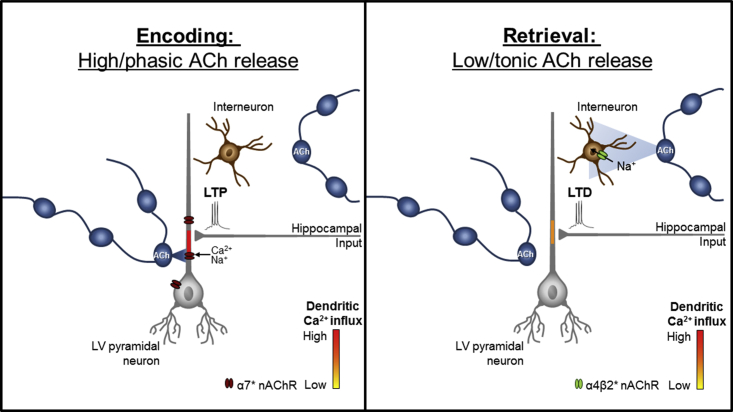
Nicotinic Modulation of Layer V Pyramidal Neurons during Encoding and Retrieval of Associative Recognition Memory Schematic representation of nAChR subtype-specific regulation of HPC-mPFC transmission during memory encoding (left) and retrieval (right) resulting from differential modes or concentrations of ACh release. During encoding, ACh is released in a phasic manner, giving rise to high concentrations that activate α7 nAChRs on pyramidal cells. This coupled with presynaptic HPC activity and pyramidal cell firing results in sufficient postsynaptic calcium to trigger LTP at the HPC-mPFC synapse. During retrieval, ACh is released in a diffuse manner giving rise to low concentrations that activate α4β2 nAChRs on interneurons. The resultant GABAergic signaling attenuates an STDP-induced increase in postsynaptic calcium levels, leading to the triggering of LTD.

Several factors could contribute to the preferential activation of nAChR subtypes during initial encoding and subsequent memory retrieval (Figure 4). High cholinergic tone promotes encoding of new information by enhancing afferent signals, while lower concentrations may favor recurrent activity and thus consolidation and retrieval (Hasselmo, 2006). Thus, during encoding, synaptic α7 nAChRs, having rapid desensitization and a high-micromolar half-activation dose for acetylcholine (ACh), can be activated by transiently high ACh concentrations released under these conditions (Hasselmo, 2006, Arroyo et al., 2014, Parikh et al., 2007, Bennett et al., 2012, Dani and Bertrand, 2007). Postsynaptic α7 nAChRs on layer V pyramidal neurons can increase calcium influx and drive pyramidal cell depolarization, while astrocytic α7 nAChRs can promote glial D-serine release (Poorthuis et al., 2013, Dani and Bertrand, 2007, Papouin et al., 2017); all of these actions promote LTP (Yakel, 2014). Furthermore, α7 nAChR depolarizing currents can inactivate transient K^+^ currents and promote back propagating action potentials to enhance STDP-LTP (Sjöström and Nelson, 2002). Our postsynaptic BAPTA data demonstrate that increases in postsynaptic calcium are critical for α7 nAChR LTP at the HPC-mPFC input. In addition, it has also been shown that in some areas, activation of presynaptic α7 nAChRs on glutamatergic terminals can promote LTP (Mansvelder and McGehee, 2000). Therefore, high ACh release during encoding most likely favors LTP of HPC-mPFC synapses through actions primarily at α7 nAChRs (Figure 4).

Lower concentrations of ACh occur during associative recognition retrieval (Hasselmo, 2006). Diffuse, tonic release of low ACh concentrations likely favors heteromeric α4β2^∗^ nAChR activation (Figure 4), which have low-micromolar effective half-activation doses and are expressed extra-synaptically (Dani and Bertrand, 2007, Arroyo et al., 2014, Bennett et al., 2012). α4β2^∗^ nAChRs in mPFC layers II/III and V are largely restricted to interneurons (Poorthuis et al., 2013, Couey et al., 2007); their activation increases GABA release onto layer V pyramidal neurons, leading to a reduction in glutamatergic driven dendritic calcium influx, thereby promoting LTD (Couey et al., 2007, Marlin and Carter, 2014, Sato et al., 2017). Our data showing that GABA antagonism prevents α4β2^∗^ nAChR LTD suggests the importance of GABAergic drive in LTD. Therefore, low ACh release during retrieval most likely favors LTD of HPC-mPFC synapses through actions primarily at α4β2^∗^ nAChRs on GABAergic interneurons (Figure 4).

Learning is associated with both LTP and LTD (Griffiths et al., 2008, Kemp and Manahan-Vaughan, 2007, Whitlock et al., 2006). Therefore, we employed the widely used peptides ZIP and the GluR2_3γ_, which prevent surface expression and endocytosis of GluA2-containing AMPA receptors, respectively (Serrano et al., 2005, Ren et al., 2013, Evuarherhe et al., 2014, Ahmadian et al., 2004, Brebner et al., 2005). While there is some debate concerning the precise molecular mechanisms by which ZIP blocks LTP (Wu-Zhang et al., 2012), our data showing that ZIP blocked LTP, but not LTD, while GluR2_3γ_ blocked LTD, but not LTP, demonstrate that each is selective for one form of plasticity. Our data therefore demonstrate that LTP is required for encoding, while LTD contributes to retrieval of associative recognition memory. While we demonstrate a role for nAChRs in both learning and in plasticity, it is nevertheless possible that encoding and retrieval may rely on some additional non-nicotinic forms of LTP and LTD. We speculate that α7 nAChR-dependent enhancement of HPC-mPFC synaptic transmission promotes and strengthens the association between items and their context during learning. α4β2^∗^ nAChR LTD at HPC-mPFC synapses may promote retrieval by reducing encoding interference from the afferent HPC input and/or increasing the signal to noise ratio of other inputs to the mPFC that are required for retrieval. A lack of temporal resolution means that learning impairments in α7 and β2 knockout mice (Picciotto et al., 2001) cannot be attributed to deficits in encoding or retrieval. Our findings now raise the possibility that selective disruption of LTP or LTD may underlie the cognitive deficits previously observed in α7 and β2 knockout mice and that deficits may be specific to encoding and retrieval, respectively (Picciotto et al., 2001).

Activation of nAChRs contributes to a wide range of cognitive functions (Dani and Bertrand, 2007, Levin et al., 2006). Our data showing that α7 and α4β2^∗^ nAChRs are required for different phases of memory, most likely through differential regulation of HPC-mPFC synaptic plasticity, highlights the complex roles that ACh plays in learning and memory. These data suggest that knowing whether memory deficits are due to deficiencies in encoding or retrieval may enable more targeted pharmacological interventions.

## Experimental Procedures

### Behavioral Procedures

All procedures were conducted in accordance with the UK Animals Scientific Procedures Act (1986) and local University of Bristol ethics regulations. A full description of methods can be found in Supplemental Experimental Procedures. In brief, mPFCs of adult male Lister Hooded rats were bilaterally cannulated (anteroposterior [AP] +3.20; medial-lateral [ML] ± 0.75; dorsoventral [DV] −3.5). OiP and OL tasks were conducted in an open field arena (Figures 1A and 1D). Rats were habituated across 5 days. In OiP, 4 distinct Duplo constructions are presented (5 min). and after 24 hr. the objects are re-presented (3 min) in a novel configuration. In OL, 2 identical Duplo objects are presented (3 min) and then re-presented (3 min) with 1 object in a novel position. Intra-mPFC infusions (1 μL/hemi, 0.5 μL/min) were timed to affect encoding, consolidation, or retrieval. Infusions and objects were counterbalanced within the experiment. Exploration was scored blind to drug condition and discrimination ratio (DR = [moved (s) − unmoved (s)]/total (s)) calculated. After experiments, rats were perfused and coronal PFC sections (40 μm) stained with cresyl violet to map cannula tip position against standard sections of rat brain (Paxinos and Watson, 1998).

### Electrophysiology

Coronal prefrontal sections (300 μm) were prepared from juvenile (30-day-old) male rats (Parent et al., 2010, Banks et al., 2015) and whole-cell recordings (K-gluconate-based internal) made from layer V pyramidal neurons. Data were collected using an Axopatch 200B amplifier (Axon Instruments) and WinLTP v1.10 software (Anderson and Collingridge, 2007). Cells were held at −70 mV (not adjusted for junction potential). Cells with series resistance >25 MΩ or variation >30% from baseline were discarded from analysis. Basal responses were evoked by extracellular stimulation of HPC input (0.1 Hz), and plasticity was induced by a spike-timing-dependent plasticity protocol (80 trains of pairings delivered at 5 Hz in current clamp), with each train at 5 excitatory postsynaptic potentials (EPSPs) (100 Hz) paired from the third EPSP to 3 postsynaptic action potentials (APs) evoked by current injection (Parent et al., 2010). Drugs were bath applied or loaded through the recording electrode, as indicated.

### Statistical Analysis

Mean DRs were compared using one-sample t tests against zero (chance). Paired two-tailed t tests compared DRs and total exploration times between vehicle and drug conditions. Mean EPSC amplitudes (5 min baseline) were compared against the final 5 min of plasticity by paired two-tailed t tests or repeated-measures ANOVA (Bonferroni corrected post hoc comparisons). Statistical analysis was conducted using raw data, and graphs are presented as means (±SEM) normalized to baseline. Significance was assumed at p < 0.05.

## Supplemental Information

Document S1. Supplemental Experimental Procedures and Tables S1–S3

Document S2. Article plus Supplemental Information
