# Supplementary material for: Nicotinic Acetylcholine Receptors Control Encoding and Retrieval of Associative Recognition Memory through Plasticity in the Medial Prefrontal Cortex
Source: Cell Rep. 2018 Mar 27;22(13):3409–15. doi: 10.1016/j.celrep.2018.03.016 (PMC5896173; doi:10.1016/j.celrep.2018.03.016)
Supplement: Document S1. Supplemental Experimental Procedures and Tables S1–S3 [file mmc1.pdf]

Cell Reports, Volume *22*

Supplemental Information

Nicotinic Acetylcholine Receptors Control Encoding and Retrieval of Associative Recognition Memory through Plasticity in the Medial Prefrontal Cortex

Marie H. Sabec, Susan Wonnacott, E. Clea Warburton, and Zafar I. Bashir

Supplemental Information

Supplemental Information Inventory:

Supplemental Tables S1-S3

Table S1. (Related to Figure 1) Total object exploration in OiP with nAChR inactivation

Table S2. (Related to Figure 1) Total object exploration in OL with nAChR inactivation

Table S3. (Related to Figure 3) Total object exploration in object-in-place task with AMPA receptor trafficking disruption

Supplemental Experimental Procedures

Supplemental References

Table S1: Total object exploration in OiP with nAChR inactivation. Related to Figure 1

Total exploration of the four objects did not differ between control or drug condition at any time point as analysed by paired t-test. Data is presented as mean ± SEM.

Infusion	Condition	Sample Phase Exploration (s)	P Value	Test Phase Exploration (s)	P Value
Pre-Sample	Vehicle	43.1 ± 3.5	0.963	29.9 ± 2.1	0.374
	MLA	42.8 ± 3.3		27.2 ± 2.6	
	Vehicle	37.4 ± 5.1	0.794	30.4 ± 2.1	0.719
	α-BGT	36.3 ± 3.1		29.2 ± 2.6	
	Vehicle	52.6 ± 6.4	0.464	40.4 ± 3.3	0.864
	DHβE	48.9 ±4.3		41.2 ± 4.6	
Post-Sample	Vehicle	63.2 ± 4.6	0.227	46.1 ± 4.4	0.361
	MLA	54.9 ± 3.4		43.2 ± 3.9	
	Vehicle	50.3 ± 4.9	0.272	34.9 ± 3.7	0.599
	DHβE	55.7 ± 2.4		37.6 ± 2.7	
Pre-Test	Vehicle	63.8 ± 3.3	0.826	45.4 ± 6.7	0.648
	MLA	65.3 ± 5.4		43.0 ± 5.4	
	Vehicle	36.4 ± 3.7	0.762	23.5 ± 2.0	0.264
	α-BGT	35.0 ± 3.7		29.5 ± 6.1	
	Vehicle	56.7 ± 4.5	0.455	36.5 ± 3.2	0.913
	DHβE	52.2 ± 3.9		37.1 ± 4.1	

Table S2: Total object exploration in OL with nAChR inactivation. Related to Figure 1

Total exploration of the two objects did not differ between control or drug condition at any time point as analysed by paired t-test. Data is presented as mean ± SEM.

Infusion	Condition	Sample Phase Exploration (s)	P Value	Test Phase Exploration (s)	P Value
Pre-Sample	Vehicle	51.4 ± 4.5	0.482	40.7 ± 2.3	0.954
	MLA	47.5 ± 3.4		41.1 ± 5.2	
Post-Sample	Vehicle	51.3 ± 3.8	0.885	30.1 ± 4.0	0.315
	DHβE	50.8 ± 4.2		35.4 ± 3.0	

Table S3: Total object exploration in object-in-place task with AMPA receptor trafficking disruption. Related to Figure 3

Total exploration of the four presented objects did not differ between active and inactive peptide conditions at any time point as analysed by paired t-test. Data is presented as mean ± SEM.

Infusion	Condition	Sample Phase Exploration (s)	P Value	Test Phase Exploration (s)	P Value
Pre-Sample	Scr-ZIP	52.2 ± 3.7	0.379	41.3 ± 4.2	0.953
	ZIP	49.0 ± 3.8		41.0 ± 3.9	
	Scr TAT-GluA2$_{3\gamma}$	58.4 ± 3.7	0.308	34.8 ± 3.7	0.130
	TAT-GluA2$_{3\gamma}$	52.8 ± 5.5		40.4 ± 4.7	
Post-Sample	Scr-ZIP	53.4 ± 4.5	0.649	33.8 ± 3.8	0.386
	ZIP	56.2 ± 3.7		38.3 ± 2.6	
	Scr TAT-GluA2$_{3\gamma}$	59.2 ± 3.9	0.837	35.8 ± 3.3	0.835
	TAT-GluA2$_{3\gamma}$	58.5 ± 4.1		35.2 ± 3.3	

Supplemental Experimental Procedures

Animals

Male Lister Hooded rats (Harlan Laboratories, UK) were used in all experiments. Surgical procedures and behavioural tests were conducted on 34 adult rats (>300g). Subjects were removed from further testing if the implanted cannula became damaged or dislodged. Rats aged post-natal day 30 were used for slice electrophysiology. All animals were group housed under a 12hour light/dark cycle (light phase 18:00-06:00), with experimental procedures conducted during the dark phase of the cycle. Water and food was available ad libitum. All procedures were conducted in accordance with the UK Animals Scientific Procedures Act (1986), in addition to local rules and regulations of the University of Bristol.

Stereotaxic Surgery

Chronically indwelling guide cannula were implanted under inhaled isoflurane anaesthetic (Induction 4%, maintenance 2-3%; Merial, Harlow, UK). Following exposure of the skull, small burr holes were drilled and stainless steel guide cannula (26 gauge; Plastics One, Semat, UK) were targeted bilaterally into the mPFC (From bregma, AP +3.20mm; ML ±0.75mm; DV -3.5mm). Implanted cannula were secured to the skull by three stainless steel screws (Plastics One, Semat, UK) and dental cement. Dummy cannulae were inserted into guide cannula and dust caps fitted to minimise risk of post-surgical infection. Saline (5ml subcutaneous) and analgesic solution (0.05ml intramuscular; Vetergesic 0.3mg/ml) were administered for hydration and pain management on completion of the surgery. Rats were single housed for 7 days following surgery and allowed 2 weeks recovery before behavioural testing.

Behavioural Procedures

Associative recognition memory was assessed using an object-in-place (OiP) task and spatial discrimination by object-location (OL) tasks (**Fig 1A,D**). Tests were conducted in an open field arena (length; 100cm, width; 90cm, height; 50cm) with global spatial cues available. A video recorder was positioned above the arena to enable recording of exploration data. All rats were habituated to handling and the arena across 5 consecutive daily sessions.

The OiP and OL tasks consist of a sample and test phase, separated by 24h retention delay. In the OiP sample phase, rats were individually positioned in the centre of the arena and permitted free exploration of four different objects for 5mins. The objects (> 9 × 8 × 7 cm) were constructed from duplo® and were presented in the corners of the arena, 15cm from the arena walls. In the test phase (3min) two of the objects were switched in position to introduce novelty in the configuration. In the OL sample phase the animals were presented with two identical duplo® objects for 3min. Following the retention delay, the animal was replaced in the arena for the test phase, in which one object was moved into a novel position within the arena.

Exploration of each object was measured in both the sample and test phases. Exploration was counted when a rat was positioned with its nose directed towards and <2cm from an object. A minimum criterion of 15secs of total exploration during the sample phase and 10secs during the test phase was required for inclusion in statistical analysis. Exploration was scored by an experimenter blind to the drug-condition and the configuration of objects presented and moved (left or right pairs) counterbalanced within each experiment.

Novelty preference is reflected in a positive discrimination ratio (DR), calculated as the difference in the time spent exploring the moved vs unmoved objects divided by the total amount of exploration. DR=0 signifies equal exploration of moved and unmoved objects, and thus chance.

Infusions
Infusions of drug or vehicle were given to a volume of 1µL per PFC hemisphere through implanted guide cannula. Infusions delivered 15mins prior to sample phase to study effects on memory acquisition, immediately after sample exploration to study effects on memory consolidation, or 15mins before the test phase to study effects on memory retrieval. In line with previous work, infusions of TAT conjugated peptides were delivered 1hour before sample or test phases (Cazakoff & Howland., 2011). A within-subjects experimental design was used with the order of treatment (i.e. drug or vehicle) counter-balanced within experiment.

Acute Slice Preparation
Rats were decapitated under isoflurane anaesthesia and the brains were rapidly transferred into ice cold cutting solution (189mM sucrose, 10mM D-glucose, 26mM NaHCO3, 3mM KCl, 5mM MgSO47H2O, 0.1mM CaCl2, 1.25mM NaH2PO4) equilibrated with carbogen (95% oxygen, 5% carbon dioxide).

Prefrontal sections (300µm) were cut using a vibratome (Campden instruments, Ltd. Model 7000 smz2). Coronal sections were cut with a 10° angle to preserve the hippocampal afferents to the mPFC, as previously described (Parent et al., 2010). Slices were incubated at room temperature in carbogenated artificial cerebrospinal fluid (aCSF; 124mM NaCl, 3mM KCl, 26mM NaHCO3, 1.25mM NaH2PO4, 2mM CaCl2, 1mM MgSO4 and 10mM D-glucose) for at least 60mins. Post-incubation, individual slices were transferred into a recording chamber and perfused with aCSF at a rate of 2ml/min at 32.0±1.0°C (Campden instruments, Ltd. Model 7800).

Electrophysiology
Prelimbic layer V pyramidal neurons were visually identified by differential interference contrast (DIC) immersion microscopy (Olympus, LUMPlanFL N, 40x lens). Data were collected using an Axopatch 200B amplifier (Axon instruments, Ltd.) and WinLTP v1.10 software (Anderson & Collingridge., 2007), then analysed off-line. Recordings were low-pass filtered at 5kHz and digitized at 20kHz. Borosilicate glass capillaries were pulled into low resistance recording electrodes (2.5-5.5MΩ) (Sutter instruments co. Model P-87) and backfilled with intracellular solution (145mM K-gluconate, 5mM NaCl, 10mM HEPES, 0.2mM EGTA, 0.3mM GTP-Na, 4mM ATP Mg; pH 7.20-7.30, 280-290mOsM). For experiments in which peptides were loaded into the cell via the recording electrode, peptidase inhibitors (0.1mM Bestatin hydrochloride, 0.1mM Leupeptin hydrochloride) were added to the intracellular solution. Experiments were conducted in whole-cell configuration voltage-clamped to -70mV, not adjusted for liquid junction potential. Cells with series resistance values of >25MΩ, or variation >30% from baseline were discarded from analysis.

Excitatory postsynaptic currents (EPSCs) were evoked through extracellular stimulation of the hippocampal fibre bundle at 0.1Hz by a concentric bipolar electrode. Plasticity at the hippocampal-mPFC input was investigated using a paradigm of spike timing-dependent plasticity induction. A stable 5min baseline was obtained within 10mins of attaining whole-cell configuration to reduce dialysis. Cells were then held in current-clamp configuration and 80 trains of pre-post pairings were delivered at theta (5Hz) frequency. Each train consisted of 5 EPSPs at 100Hz, paired with a 7.5ms delay from the 3rd EPSP to 3 postsynaptic action potentials (APs) evoked by suprathreshold current injections (Parent et al., 2010).

Drugs
Compounds infused *in vivo* were dissolved in sterile 0.9% saline solution. Drugs used *in vitro* were dissolved in aCSF and bath applied or added to the intracellular solution for postsynaptic loading. Nicotine ditartrate, PNU-282987, RJR-2403 Oxalate, and Methyllycaconitine (MLA) were purchased

from Abcam (Cambridge Science Park, UK). Dihydro-β-erythroidine hydrobromide (DHβE), Zeta inhibitory peptide (ZIP; SIYRRGARRWRKL) and scrambled ZIP (RLYRKRIWRSAGR) were purchased from Tocris (Bristol, UK). The peptides TAT-GluR$_{3γ}$ (YGRKKRRQRRRYKEGYNVTG), scrambled TAT-GluR2$_{3γ}$ (YGRKKRRQRRRVYKYGGYNE), GluR2$_{3γ}$ (YKEGYNVYG) and GluR2$_{3A}$ (AKEGANVAG) were purchased from Eurogentec (LIEGE Science Park, Belgium. *In vivo* GluR2$_{3γ}$ and its scrambled peptide control were delivered attached to the transduction domain of the TAT protein to enable cell permeability (Brebner et al., 2005). GluR2$_{3A}$ was used for postsynaptic infusion as scrambled GluR2$_{3γ}$ was not commercially available without a conjugated TAT domain.

Statistical Analysis

Discrimination ratios (DR) were used to analyse performance in all behavioural experiments. The mean DR for each condition were compared using one-sample two-tailed t-tests against zero (chance) discrimination. Paired two-tailed t-tests were used to compare DRs between vehicle and drug conditions in individual animals. Paired t-tests were also used to compare the total time spent exploring objects in both sample and test phases of the tasks between vehicle and drug conditions.

For *in vitro* electrophysiology, the mean peak amplitude of responses recorded in the last 5mins of the baseline were compared against that of the final 5mins of plasticity by paired two-tail t-tests or repeated-measures ANOVA with Bonferroni corrected *post-hoc* comparisons. Unpaired two-tailed t-tests were used to compare the normalised response amplitudes following post-synaptic loading of active and inactive peptide. Except as stated, statistical analysis was conducted using the raw data collected, graphs are presented as mean values (±SEM) normalised to the baseline. All statistical analysis was performed using IBM SPSS 21 software package, with statistical significance was taken as $P<0.05$.

Supplemental References

Anderson, W.W., and Collingridge, G.L. (2007) Capabilities of the WinLTP data acquisition program extending beyond basic LTP experimental functions. J Neurosci Methods *162*, 346-356.

Brebner, K., Wong, T.P., Liu, L.D., Liu, Y.T., Campsall, P., Gray, S., Phelps, L., Phillips, A.G., and Wang, Y.T. (2005). Nucleus accumbens long-term depression and the expression of behavioral sensitization. Science *310* 1340-1343.

Cazakoff, B.N., and Howland, J.G. (2011) AMPA receptor endocytosis in rat perirhinal cortex underlies retrieval of object memory. Learning & Memory *18*, 688-692.

Parent, M.A., Wang, L., Su, J.J., Netoff, T. & Yuan, L.L. (2010) Identification of the hippocampal input to medial prefrontal cortex in vitro. Cereb Cortex *20*, 393-403.
