# Supplementary material for: Nicotinic Acetylcholine Receptors Control Encoding and Retrieval of Associative Recognition Memory through Plasticity in the Medial Prefrontal Cortex
Source: Cell Rep. 2018 Mar 27;22(13):3409–15. doi: 10.1016/j.celrep.2018.03.016 (PMC5896173; doi:10.1016/j.celrep.2018.03.016)
Supplement: Document S2. Article plus Supplemental Information [file mmc2.pdf]

Cell Reports

Report

Nicotinic Acetylcholine Receptors Control Encoding and Retrieval of Associative Recognition Memory through Plasticity in the Medial Prefrontal Cortex

Graphical Abstract

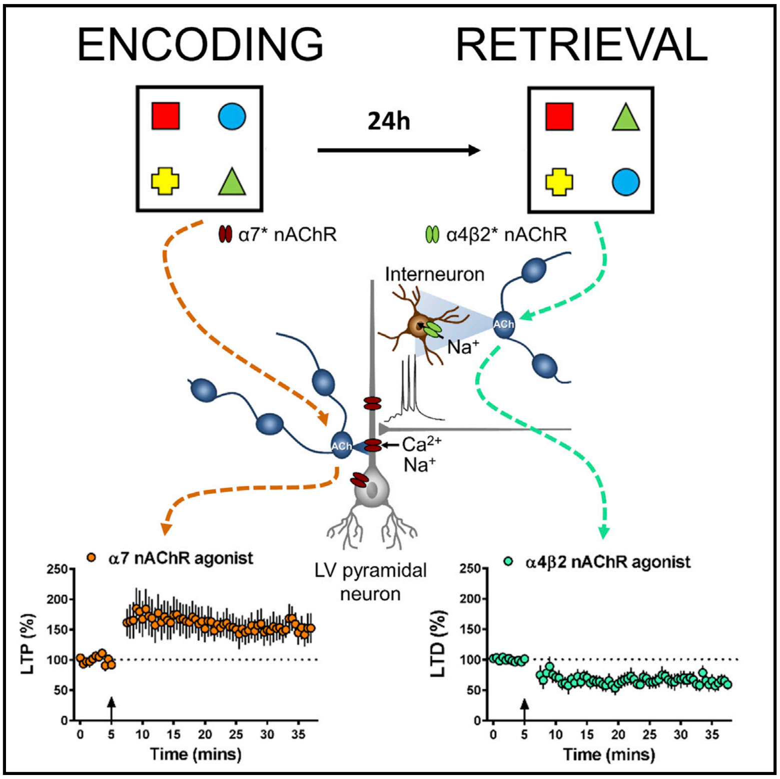

Authors

Marie H. Sabec, Susan Wonnacott, E. Clea Warburton, Zafar I. Bashir

Correspondence

marie.sabec@pasteur.fr

In Brief

Sabec et al. reveal a divergence in function of prefrontal nicotinic receptor subtypes in different stages of long-term associative recognition memory that relates to bidirectional modulation of synaptic plasticity at hippocampal-prefrontal synapses.

Highlights

- Prefrontal α7 nAChRs are critical for encoding of associative recognition memory
- Prefrontal α4β2 nAChRs are required for retrieval of associative recognition memory
- α7 and α4β2 nAChRs gate bidirectional plasticity at hippocampal-prefrontal synapses
- Bidirectional plasticity underlies the role of nAChR in associative recognition

Sabec et al., 2018, Cell Reports *22*, 3409–3415
March 27, 2018 © 2018 The Authors.
https://doi.org/10.1016/j.celrep.2018.03.016

CellPress

Cell Reports
Report

OPEN ACCESS
CellPress

Nicotinic Acetylcholine Receptors Control Encoding and Retrieval of Associative Recognition Memory through Plasticity in the Medial Prefrontal Cortex

Marie H. Sabec,[1,3,4,*] Susan Wonnacott,[2] E. Clea Warburton,[1] and Zafar I. Bashir[1]
[1]School of Physiology, Pharmacology and Neuroscience, University of Bristol, Bristol BS8 1TD, UK
[2]Department of Biology and Biochemistry, University of Bath, Bath BA2 7AY, UK
[3]Present address: Department of Neuroscience, Pasteur Institute, Paris 75015, France
[4]Lead Contact
*Correspondence: marie.sabec@pasteur.fr
https://doi.org/10.1016/j.celrep.2018.03.016

SUMMARY

Nicotinic acetylcholine receptors (nAChRs) expressed in the medial prefrontal cortex have critical roles in cognitive function. However, whether nAChRs are required for associative recognition memory and the mechanisms by which nAChRs may contribute to mnemonic processing are not known. We demonstrate that nAChRs in the prefrontal cortex exhibit subtype-specific roles in associative memory encoding and retrieval. We present evidence that these separate roles of nAChRs may rely on bidirectional modulation of plasticity at synaptic inputs to the prefrontal cortex that are essential for associative recognition memory.

INTRODUCTION

Associative visual recognition is the ability to integrate the identity of an object with the location in which it was encountered (Dickerson and Eichenbaum, 2010). Associative recognition memory consists of initial encoding and subsequent retrieval and depends on the medial prefrontal cortex (mPFC) integrating information received from the hippocampus (HPC) and other brain regions (Barker et al., 2007, 2017).

Acetylcholine is essential for a variety of complex behaviors such as the performance of attention and learning tasks (Wallace and Bertrand, 2013; Parikh et al., 2007), and cholinergic deficits are central to the etiology of dementias (Picciotto and Zoli, 2002). To date, there has been a focus on muscarinic acetylcholine receptors in mPFC-dependent memory (Barker and Warburton, 2008). However, it is not known whether nicotinic acetylcholine receptors (nAChRs) in the mPFC play any role in encoding, consolidation, or retrieval of associative recognition memory in rats.

Synaptic plasticity is considered essential for learning and memory (Martin et al., 2000). nAChRs are expressed throughout the mPFC (Poorthuis et al., 2013), and their activation can give rise to synaptic plasticity (Verhoog et al., 2016; Couey et al., 2007; Udakis et al., 2016). HPC input to the mPFC is crucial for associative recognition memory (Barker et al., 2017), but whether activation of nAChRs governs synaptic plasticity at HPC-mPFC synapses and how such nicotinic modulation may be involved in distinct phases of associative recognition memory are not known.

We now test the hypothesis that specific nAChRs induce different forms of synaptic plasticity to bring about encoding and retrieval of associative recognition memory. We report that homomeric $\alpha7$ nAChRs are essential for both encoding of associative recognition and induction of long-term potentiation (LTP) of HPC-mPFC synapses. In contrast, $\alpha4\beta2$-containing ($\alpha4\beta2^*$) nAChRs are essential for both retrieval of associative memory and long-term depression (LTD). Selective inhibition of LTP or LTD expression mechanisms prevented memory encoding and retrieval, respectively. We conclude that different nAChRs in the mPFC promote LTP or LTD of HPC-mPFC synapses to enable encoding or retrieval of associative recognition memory.

RESULTS

$\alpha7$ nAChRs Are Required for Encoding and $\alpha4\beta2$ nAChRs for Retrieval of Associative Recognition Memory

Selective antagonists of $\alpha7$ and $\alpha4\beta2^*$ receptors were infused intra-cortically into mPFC during different phases of the object-in-place (OiP) task (Figure 1A). The $\alpha7$ nAChR antagonist methyllycaconitine citrate (MLA) (100 nM), when given prior to the sample phase, impaired OiP discrimination (MLA versus vehicle [Veh] $t_{(10)} = 2.756$, $p = 0.021$). Thus, following MLA, discrimination was not different from chance, while the vehicle group had a significant discrimination (MLA $t_{(10)} = 0.372$, $p = 0.717$; Veh $t_{(10)} = 6.368$, $p < 0.001$; Figure 1Bi). In contrast, there were no deficits in OiP when MLA or vehicle was delivered after the sample phase (MLA $t_{(11)} = 3.335$, $p = 0.007$; Veh $t_{(11)} = 4.382$, $p = 0.001$; MLA versus Veh $t_{(11)} = 0.820$, $p = 0.429$; Figure 1Bi) or prior to the test phase (MLA $t_{(9)} = 5.559$, $p < 0.001$; Veh $t_{(9)} = 4.145$, $p = 0.003$; MLA versus Veh $t_{(9)} = 0.190$, $p = 0.854$; Figure 1Bi).

To confirm these effects, the experiment was repeated with α-bungarotoxin (α-BGT; 1 μM). Infusion of α-BGT prior to the sample phase impaired OiP encoding (α-BGT versus Veh

This is an open access article under the CC BY license (http://creativecommons.org/licenses/by/4.0/).

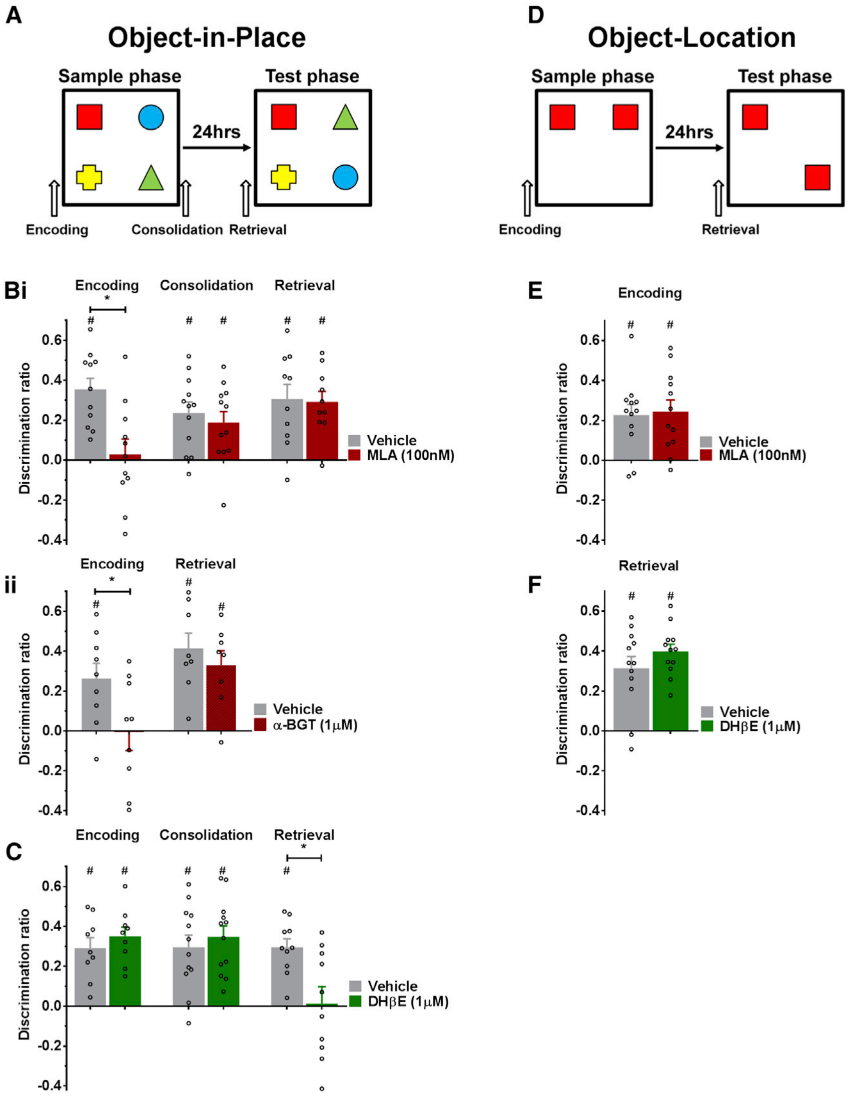

Figure 1. Associative Recognition Memory Is Dependent on Activation of Different nAChRs in the mPFC

(A) Schematic of OiP task. Arrows represent the timing of mPFC infusions.

(B) MLA impaired OiP memory when infused before the sample phase (encoding; n = 11), but not after the sample phase (consolidation; n = 12) or before the test phase (retrieval; n = 10) (i). α-BGT also impaired discrimination when infused before the sample phase (n = 9), but not before the test phase (n = 8) (ii).

(C) DHβE impaired OiP memory if given before the test phase (n = 10), but not before (n = 9) or after (n = 12) the sample phase.

(D) Schematic of the OL task

(E and F) OL memory was not impaired by MLA infusion before the sample phase (n = 12) (E) or DHβE infusion before the test phase (n = 12) (F).

Data are presented as mean ± SEM (*p < 0.05 paired t test; #p < 0.05 one-sample t test against 0). See also Tables S1 and S2.

$t_{(8)} = 2.559$, $p = 0.034$), and animals failed to discriminate (α-BGT $t_{(8)} = -0.075$, $p = 0.942$; Veh t(8) = 3.419, $p = 0.009$; Figure 1Bii). There was no deficit in OiP when α-BGT or vehicle was delivered prior to the test phase (α-BGT $t_{(7)} = 4.601$, $p = 0.002$; Veh; $t_{(7)} = 5.360$, $p = 0.001$; α-BGT versus Veh $t_{(7)} = 1.276$, $p = 0.243$; Figure 1Bii). Thus, α7 nAChRs in mPFC are critical for encoding, but not for consolidation or retrieval of long-term associative recognition memory.

Infusion of the α4β2* nAChR antagonist DHβE (1 μM) impaired discrimination when given prior to the test phase (DHβE $t_{(9)} = 0.141$, $p = 0.891$; Veh $t_{(9)} = 6.954$, $p < 0.001$; Figure 1C); there was a significant difference between DHβE and vehicle (DHβE versus Veh $t_{(9)} = 2.467$, $p = 0.036$). Memory was not impaired following administration of DHβE either prior to the sample phase (DHβE $t_{(8)} = 7.643$, $p < 0.001$; Veh $t_{(8)} = 5.593$, $p = 0.001$; DHβE versus Veh $t_{(8)} = -1.085$, $p = 0.310$) or after the sample phase (DHβE $t_{(11)} = 6.342$, $p < 0.001$; Veh $t_{(11)} = 4.831$, $p = 0.001$; DHβE versus Veh $t_{(11)} = 0.\text{-}606$, $p = 0.557$; Figure 1C). Thus, α4β2* nAChRs in the mPFC are critical for the retrieval of long-term associative recognition memory, but not for its consolidation or encoding.

There was no difference in total object exploration during sample or test phases during either α7 nAChR or α4β2* nAChR antagonism (Table S1), indicating the drugs had no effect on motor function or exploratory behavior.

To ensure effects of nAChR inhibition were not due to deficits in attention (Wallace and Bertrand, 2013), animals were tested on a non-associative object location (OL) task (Figure 1D) that is independent of the mPFC (Barker et al., 2007). Infusion of MLA prior to sample phase or DHβE prior to test phase had no effect on OL memory (Figures 1E and 1F; MLA $t_{(11)} = 4.220$, $p = 0.001$; Veh $t_{(11)} = 4.263$, $p = 0.001$; MLA versus Veh $t_{(11)} = -0.236$, $p = 0.818$; DHβE $t_{(11)} = 11.193$, $p < 0.001$; Veh $t_{(11)} = 5.366$, $p < 0.001$; DHβE versus Veh $t_{(11)} = -1.188$, $p = 0.260$). In addition, neither MLA nor DHβE had any effect on total exploration times (Table S2).

α7 and α4β2 nAChRs Are Required for LTP and LTD, Respectively

To probe how nAChRs may contribute to separate phases of associative recognition we examined, *in vitro*, synaptic plasticity at the HPC-mPFC pathway (Banks et al., 2015) that is essential for OiP memory (Barker et al., 2017). A spike-timing-dependent plasticity protocol (STDP; Parent et al., 2010) resulted only in a transient increase in HPC-mPFC EPSCs ($t_{(7)} = -0.410$, $p = 0.694$; Figures 2A and 2I). To test whether nAChR subtypes can regulate synaptic plasticity, selective agonists were applied with STDP. In the presence

OPEN ACCESS
CellPress

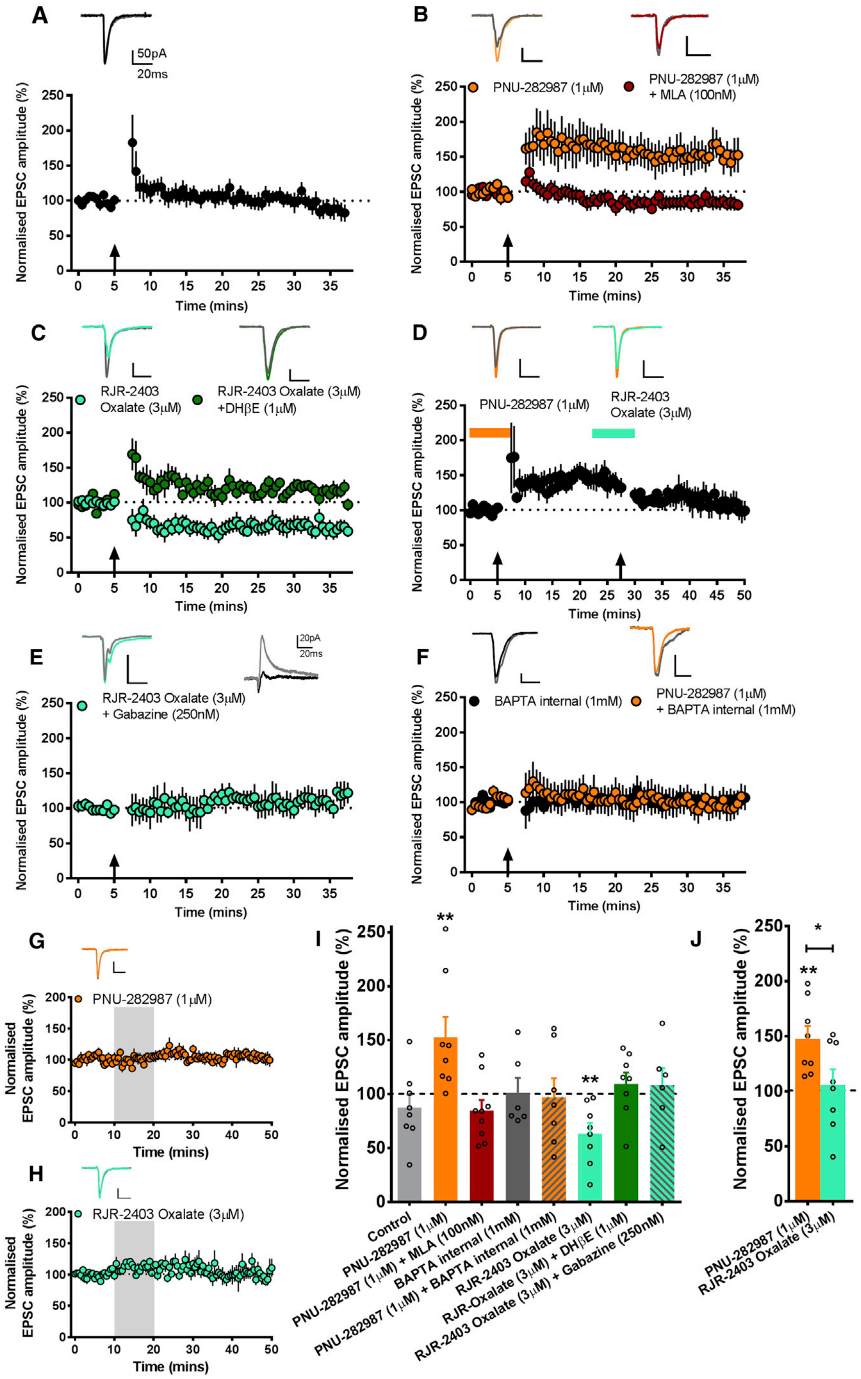

Figure 2. nAChRs Bidirectionally Modulate HPC-mPFC Plasticity

(A) Transient potentiation following delivery of STDP, indicated by arrow (n = 8).

(B) LTP induced by combined STDP and PNU-282987 (n = 8) was blocked in separate experiments by co-application of MLA (n = 9).

(C) LTD induced by STDP with RJR-2403 oxalate (n = 8) was blocked by co-application of DHβE (n = 8).

(D) Induction of PNU-282987 STDP LTP was reversed by RJR-2403 Oxalate STDP induced LTD (n = 8).

(E and F) α4β2 nAChR LTD was blocked in the presence of gabazine (n = 6) (E), and α7 nAChR LTP was blocked by postsynaptic BAPTA (n = 7) (F).

(G and H) Application of PNU-282987 (n = 6) (G) or RJR-2403 oxalate (n = 6) (H) in the absence of STDP did not induce plasticity. In all graphs, representative EPSCs are shown from baseline (gray traces) and the last 5 min (colored traces) of the experiment.

(I and J) Summary of normalized EPSC amplitudes recorded in the final 5 min of each STDP experiment.

Data are presented as normalized mean ± SEM (*$p < 0.05$, **$p < 0.01$; paired t test in I or repeated-measures ANOVA with Bonferroni post hoc in J).

of the α7 nAChR agonist PNU-282987 (1 μM) LTP was induced by STDP ($t_{(7)} = 4.059$, $p = 0.005$; Figures 2B and 2I). LTP was prevented by co-application of α7 nAChR antagonist MLA (100 nM) ($t_{(8)} = -1.583$, $p = 0.152$; Figures 2B and 2I) or intracellular 1,2-bis(o-aminophenoxy)ethane-N,N,N′,N′-tetra-acetic acid (BAPTA) (1 mM) ($t_{(6)} = -0.237$, $p = 0.821$; control $t_{(5)} = -0.708$, $p = 0.510$; Figures 2F and 2I).

LTD was induced when STDP was delivered in the presence of the α4β2* agonist RJR-2403 oxalate (3 μM) ($t_{(7)} = -4.518$, $p = 0.003$; Figures 2C and 2I); LTD was prevented by co-application of the α4β2* antagonist DHβE (1 μM) ($t_{(7)} = 1.100$, $p = 0.308$; Figures 2C and 2I). Interestingly, α4β2* nAChR activation coupled with STDP reversed prior α7 nAChR induced LTP ($F_{(2,14)} = 8.963$, $p = 0.003$; Figures 2D and 2J). Moreover, LTD was blocked in the presence of gabazine (250 nM) ($t_{(5)} = 0.651$, $p = 0.544$; Figures 2E and 2I), suggesting GABAergic transmission is required for LTD induction.

Neither α7 nor α4β2* nAChR agonists affected synaptic transmission in the absence of STDP (PNU $t_{(5)} = -0.489$, $p = 0.645$; RJR $t_{(5)} = -1.126$, $p = 0.311$; Figures 2G and 2H). Together, these results demonstrate that paired pre- and postsynaptic activity combined with α7 or α4β2* nAChR activation differentially induces LTP and LTD at the HPC-mPFC pathway. LTP may rely on α7 nAChR-mediated increases in intracellular calcium and LTD on α4β2* nAChR-mediated GABAergic inhibition.

Expression of α7 nAChR LTP Is Dependent on Atypical PKCs, while α4β2 nAChR LTD Requires GluA2 Internalization

To test whether bidirectional plasticity may provide a mechanism by which different nAChR subtypes contribute to associative recognition, we first determined whether α7 and α4β2*

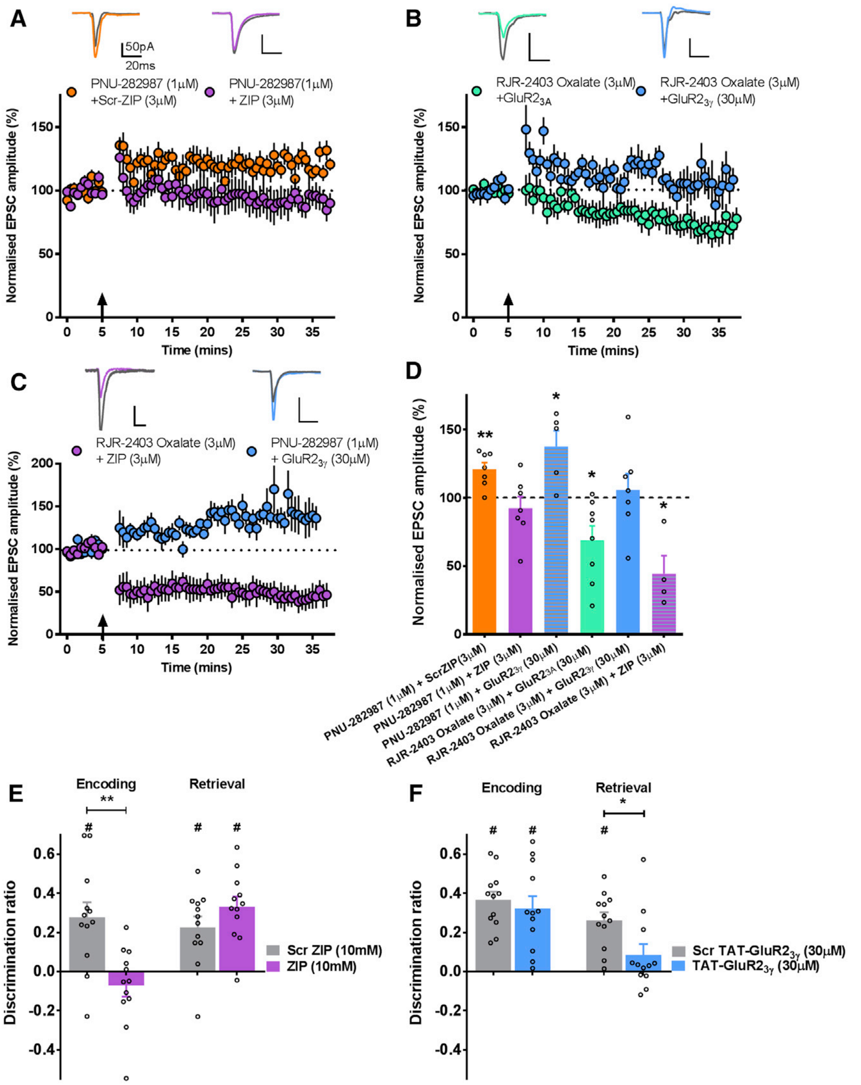

Figure 3. Disrupting Expression of LTP or LTD Blocks α7-Induced LTP and α4β2-Induced LTD and Blocks Associative Memory Encoding and Retrieval, Respectively

(A) ZIP (n = 7), but not Scr-ZIP (n = 7), prevented STDP/PNU-282987 induction of LTP.

(B) GluR2$_{3\gamma}$ (n = 7), but not GluR2$_{3A}$ (n = 8), prevented STDP/DHβE induction of LTD.

(C) α7 nAChR LTP was not blocked by GluR2$_{3\gamma}$ (n = 5), and α4β2 nAChR LTD was not blocked by ZIP (n = 4). Representative traces shown from baseline and the last 5 min of the experiment.

(D) Summary of normalized EPSC amplitudes recorded in the final 5 min of each experiment.

(E) ZIP impaired OiP memory when infused before the sample phase (n = 12), but not the test phase (n = 12).

(F) TAT-GluR2$_{3\gamma}$ impaired OiP when infused before the test phase (n = 12), but not the sample phase (n = 12). The average discrimination following two trials with TAT-GluR2$_{3\gamma}$/Scr TAT-GluR2$_{3\gamma}$ infusion before the test phase is shown.

Data are presented as mean ± SEM (*p < 0.05, **p < 0.01 paired t test; #p < 0.05 one-sample t test against 0). See also Table S3.

nAChR-gated plasticity could be blocked by selective manipulation of intracellular mechanisms that mediate LTP or LTD expression. Zeta inhibitory peptide (ZIP) blocks LTP through inhibition of atypical PKC isoforms PKCι/λ and PKMζ (Serrano et al., 2005; Ren et al., 2013). Loading mPFC pyramidal cells with ZIP (3 μM) via the recording electrode blocked α7 nAChR-dependent LTP ($t_{(6)} = -0.975$, p = 0.367; Figures 3A and 3D). In contrast, α7 nAChR-LTP was not blocked by postsynaptic loading of scrambled ZIP ($t_{(6)} = 4.047$, p = 0.007; Figures 3A and 3D)

To test expression mechanisms of α4β2* nAChR-induced LTD, we used the peptide GluR2$_{3\gamma}$ (30 μM) to inhibit activity-driven endocytosis without affecting basal transmission or LTP (Ahmadian et al., 2004; Brebner et al., 2005). Postsynaptic loading of GluR2$_{3\gamma}$ blocked α4β2* nAChR-dependent LTD ($t_{(6)} = 0.470$, p = 0.655; Figures 3B and 3D). LTD was not blocked by the inactive peptide, GluR2$_{3A}$ ($t_{(7)} = -3.215$, p = 0.015; Figures 3B and 3D)

α7 nAChR-induced LTP was not affected by GluR2$_{3\gamma}$ (30 μM) ($t_{(4)} = 3.290$, p = 0.030; Figures 3C and 3D), and α4β2* nAChR-dependent LTD was not affected by ZIP (3 μM) ($t_{(3)} = -4.504$, p = 0.020; Figures 3C and 3D), thus confirming the selectivity of the GluR2$_{3\gamma}$ for LTD and ZIP for LTP.

Blocking Expression Mechanisms of LTP and LTD Prevents Encoding and Retrieval of Associative Recognition Memory, Respectively

We next tested the hypothesis that if the different forms of nAChR-induced plasticity are essential for the separate phases of associative recognition memory, then selective blockade of LTP and LTD expression mechanisms *in vivo* should result in selective deficits in encoding and retrieval, respectively.

Infusion of ZIP (10 mM) prior to the sample phase impaired OiP performance compared to scrambled ZIP (Scr-ZIP; 10 mM) (ZIP versus Scr-ZIP $t_{(11)} = 3.293$, p = 0.004); discrimination following ZIP was not different from chance (ZIP $t_{(11)} = -1.189$, p = 0.260; Scr-ZIP $t_{(11)} = 3.622$, p = 0.004; Figure 3E). In contrast, memory was not impaired when ZIP was infused prior to the test phase (ZIP $t_{(11)} = 6.491$, p < 0.001; Scr-ZIP $t_{(11)} = 4.095$, p = 0.002; ZIP versus Scr-ZIP $t_{(11)} = -1.553$, p = 0.149; Figure 3E). Therefore, selective blockade of LTP in the mPFC causes a deficit in associative recognition memory encoding but is without effect on memory retrieval.

Delivery of TAT-GluR2$_{3\gamma}$ (30 μM) (Cazakoff and Howland, 2011) or control peptide (Scr TAT-GluR2$_{3\gamma}$; 30 μM) had no effect on OiP discrimination when delivered prior to the sample phase (TAT-GluR2$_{3\gamma}$ $t_{(11)} = 5.204$, p < 0.001; Scr TAT-GluR2$_{3\gamma}$ $t_{(11)} = 8.670$, p < 0.001; TAT-GluR2$_{3\gamma}$ versus Scr TAT-GluR2$_{3\gamma}$ $t_{(11)} = 0.716$, p = 0.489; Figure 3F). In contrast, infusions given

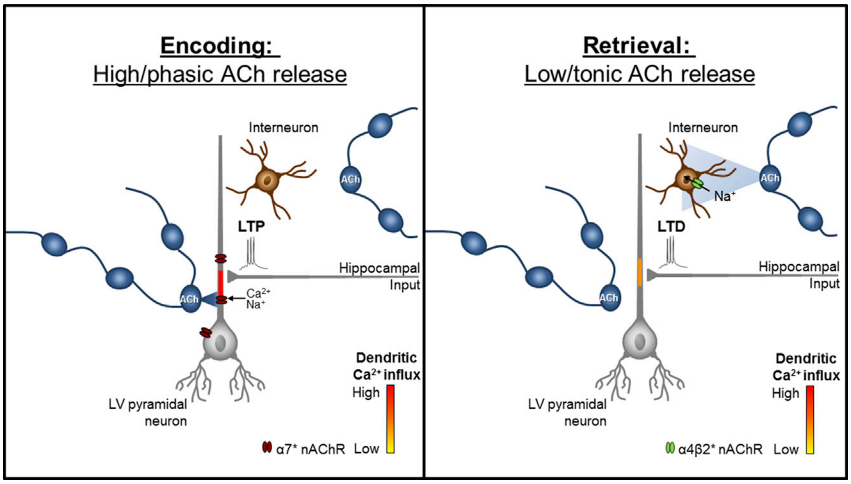

Figure 4. Nicotinic Modulation of Layer V Pyramidal Neurons during Encoding and Retrieval of Associative Recognition Memory

Schematic representation of nAChR subtype-specific regulation of HPC-mPFC transmission during memory encoding (left) and retrieval (right) resulting from differential modes or concentrations of ACh release. During encoding, ACh is released in a phasic manner, giving rise to high concentrations that activate α7 nAChRs on pyramidal cells. This coupled with presynaptic HPC activity and pyramidal cell firing results in sufficient postsynaptic calcium to trigger LTP at the HPC-mPFC synapse. During retrieval, ACh is released in a diffuse manner giving rise to low concentrations that activate α4β2 nAChRs on interneurons. The resultant GABAergic signaling attenuates an STDP-induced increase in postsynaptic calcium levels, leading to the triggering of LTD.

before the test phase produced a significant difference in memory performance between conditions (TAT-GluR2$_{3\gamma}$ versus Scr TAT-GluR2$_{3\gamma}$ $t_{(11)} = 2.251$, $p = 0.046$). Thus performance under TAT-GluR2$_{3\gamma}$ did not differ from chance (TAT-GluR2$_{3\gamma}$ $t_{(11)} = 1.527$, $p = 0.115$) in contrast to control performance (Scr TAT-GluR2$_{3\gamma}$ $t_{(11)} = 6.423$, $p < 0.001$; Figure 3F). Selective blockade of LTD in the mPFC therefore causes a deficit in long-term associative recognition memory retrieval but is without effect on memory encoding.

The deficits in memory resulting from blocking expression of plasticity were not a result of motor or attentional impairment, as total object exploration was equivalent between active and inactive peptide conditions for both ZIP and GluR2$_{3\gamma}$ (Table S3). Therefore, blockade of LTP (dependent on α7 nAChR) and LTD (dependent on α4β2* nAChR) resulted in selective deficits in the encoding and retrieval, respectively, of OiP memory.

DISCUSSION

Our study takes advantage of the temporal specificity of pharmacological interventions to enable transient receptor inactivation and demonstrates that homomeric α7 and heteromeric α4β2* nAChR subtypes make differential contributions to cognitive functions and to underlying synaptic plasticity at the HPC-mPFC pathway. These results suggest that different nAChRs promote LTP or LTD to enable encoding or retrieval of associative recognition memory.

Within the mPFC, α7 and α4β2* subtypes of nAChRs display differential expression across distinct cells and layers (Couey et al., 2007; Poorthuis et al., 2013; Verhoog et al., 2016; Wallace and Bertrand., 2013). In the current work, we focus on HPC input to pyramidal cells in layer V of the mPFC, since this input is crucial for associative recognition memory (Barker et al., 2017).

Several factors could contribute to the preferential activation of nAChR subtypes during initial encoding and subsequent memory retrieval (Figure 4). High cholinergic tone promotes encoding of new information by enhancing afferent signals, while lower concentrations may favor recurrent activity and thus consolidation and retrieval (Hasselmo, 2006). Thus, during encoding, synaptic α7 nAChRs, having rapid desensitization and a high-micromolar half-activation dose for acetylcholine (ACh), can be activated by transiently high ACh concentrations released under these conditions (Hasselmo, 2006; Arroyo et al., 2014; Parikh et al., 2007; Bennett et al., 2012; Dani and Bertrand, 2007). Postsynaptic α7 nAChRs on layer V pyramidal neurons can increase calcium influx and drive pyramidal cell depolarization, while astrocytic α7 nAChRs can promote glial D-serine release (Poorthuis et al., 2013; Dani and Bertrand, 2007; Papouin et al., 2017); all of these actions promote LTP (Yakel, 2014). Furthermore, α7 nAChR depolarizing currents can inactivate transient K^+ currents and promote back propagating action potentials to enhance STDP-LTP (Sjöström and Nelson, 2002). Our postsynaptic BAPTA data demonstrate that increases in postsynaptic calcium are critical for α7 nAChR LTP at the HPC-mPFC input. In addition, it has also been shown that in some areas, activation of presynaptic α7 nAChRs on glutamatergic terminals can promote LTP (Mansvelder and McGehee, 2000). Therefore, high ACh release during encoding most likely favors LTP of HPC-mPFC synapses through actions primarily at α7 nAChRs (Figure 4).

Lower concentrations of ACh occur during associative recognition retrieval (Hasselmo, 2006). Diffuse, tonic release of low ACh concentrations likely favors heteromeric α4β2* nAChR activation (Figure 4), which have low-micromolar effective half-activation doses and are expressed extra-synaptically (Dani and Bertrand, 2007; Arroyo et al., 2014; Bennett et al., 2012). α4β2* nAChRs in mPFC layers II/III and V are largely restricted to interneurons (Poorthuis et al., 2013; Couey et al., 2007); their activation increases GABA release onto layer V pyramidal neurons, leading to a reduction in glutamatergic driven dendritic calcium influx, thereby promoting LTD (Couey et al., 2007; Marlin and Carter, 2014; Sato et al., 2017). Our data showing that GABA antagonism prevents α4β2* nAChR LTD suggests the importance of GABAergic drive in LTD. Therefore, low ACh release during retrieval most likely favors LTD of HPC-mPFC synapses through actions primarily at α4β2* nAChRs on GABAergic interneurons (Figure 4).

Learning is associated with both LTP and LTD (Griffiths et al., 2008; Kemp and Manahan-Vaughan, 2007; Whitlock et al., 2006). Therefore, we employed the widely used peptides ZIP and the GluR2$_{3\gamma}$, which prevent surface expression and endocytosis of GluA2-containing AMPA receptors, respectively (Serrano et al., 2005; Ren et al., 2013; Evuarherhe et al., 2014; Ahmadian et al., 2004; Brebner et al., 2005). While there is some debate concerning the precise molecular mechanisms by which ZIP blocks LTP (Wu-Zhang et al., 2012), our data showing that ZIP blocked LTP, but not LTD, while GluR2$_{3\gamma}$ blocked LTD, but not LTP, demonstrate that each is selective for one form of plasticity. Our data therefore demonstrate that LTP is required for encoding, while LTD contributes to retrieval of associative recognition memory. While we demonstrate a role for nAChRs in both learning and in plasticity, it is nevertheless possible that encoding and retrieval may rely on some additional non-nicotinic forms of LTP and LTD. We speculate that α7 nAChR-dependent enhancement of HPC-mPFC synaptic transmission promotes and strengthens the association between items and their context during learning. α4β2* nAChR LTD at HPC-mPFC synapses may promote retrieval by reducing encoding interference from the afferent HPC input and/or increasing the signal to noise ratio of other inputs to the mPFC that are required for retrieval. A lack of temporal resolution means that learning impairments in α7 and β2 knockout mice (Picciotto et al., 2001) cannot be attributed to deficits in encoding or retrieval. Our findings now raise the possibility that selective disruption of LTP or LTD may underlie the cognitive deficits previously observed in α7 and β2 knockout mice and that deficits may be specific to encoding and retrieval, respectively (Picciotto et al., 2001).

Activation of nAChRs contributes to a wide range of cognitive functions (Dani and Bertrand, 2007; Levin et al., 2006). Our data showing that α7 and α4β2* nAChRs are required for different phases of memory, most likely through differential regulation of HPC-mPFC synaptic plasticity, highlights the complex roles that ACh plays in learning and memory. These data suggest that knowing whether memory deficits are due to deficiencies in encoding or retrieval may enable more targeted pharmacological interventions.

EXPERIMENTAL PROCEDURES

Behavioral Procedures

All procedures were conducted in accordance with the UK Animals Scientific Procedures Act (1986) and local University of Bristol ethics regulations. A full description of methods can be found in Supplemental Experimental Procedures. In brief, mPFCs of adult male Lister Hooded rats were bilaterally cannulated (anteroposterior [AP] +3.20; medial-lateral [ML] ± 0.75; dorsoventral [DV] −3.5). OiP and OL tasks were conducted in an open field arena (Figures 1A and 1D). Rats were habituated across 5 days. In OiP, 4 distinct Duplo constructions are presented (5 min). and after 24 hr. the objects are re-presented (3 min) in a novel configuration. In OL, 2 identical Duplo objects are presented (3 min) and then re-presented (3 min) with 1 object in a novel position. Intra-mPFC infusions (1 μL/hemi, 0.5 μL/min) were timed to affect encoding, consolidation, or retrieval. Infusions and objects were counterbalanced within the experiment. Exploration was scored blind to drug condition and discrimination ratio (DR = [moved (s) − unmoved (s)]/total (s)) calculated. After experiments, rats were perfused and coronal PFC sections (40 μm) stained with cresyl violet to map cannula tip position against standard sections of rat brain (Paxinos and Watson, 1998).

Electrophysiology

Coronal prefrontal sections (300 μm) were prepared from juvenile (30-day-old) male rats (Parent et al., 2010; Banks et al., 2015) and whole-cell recordings (K-gluconate-based internal) made from layer V pyramidal neurons. Data were collected using an Axopatch 200B amplifier (Axon Instruments) and WinLTP v1.10 software (Anderson and Collingridge, 2007). Cells were held at −70 mV (not adjusted for junction potential). Cells with series resistance >25 MΩ or variation >30% from baseline were discarded from analysis. Basal responses were evoked by extracellular stimulation of HPC input (0.1 Hz), and plasticity was induced by a spike-timing-dependent plasticity protocol (80 trains of pairings delivered at 5 Hz in current clamp), with each train at 5 excitatory postsynaptic potentials (EPSPs) (100 Hz) paired from the third EPSP to 3 postsynaptic action potentials (APs) evoked by current injection (Parent et al., 2010). Drugs were bath applied or loaded through the recording electrode, as indicated.

Statistical Analysis

Mean DRs were compared using one-sample t tests against zero (chance). Paired two-tailed t tests compared DRs and total exploration times between vehicle and drug conditions. Mean EPSC amplitudes (5 min baseline) were compared against the final 5 min of plasticity by paired two-tailed t tests or repeated-measures ANOVA (Bonferroni corrected post hoc comparisons). Statistical analysis was conducted using raw data, and graphs are presented as means (±SEM) normalized to baseline. Significance was assumed at $p < 0.05$.

SUPPLEMENTAL INFORMATION

Supplemental Information includes Supplemental Experimental Procedures and three tables and can be found with this article online at https://doi.org/10.1016/j.celrep.2018.03.016.

ACKNOWLEDGMENTS

We thank Paul Banks (P.B.) and Gareth Barker (G.B.) for assistance with experiments and P.B., G.B., and Jack Mellor for critical comments on the manuscript. This work was supported by the Biotechnology and Biological Sciences Research Council (BBSRC) (studentship BB/J014400/1 and grant BB/L001896/1) and the Wellcome Trust (grant 206401/Z/17/Z).

AUTHOR CONTRIBUTIONS

Z.I.B., M.H.S., E.C.W., and S.W. designed experiments. M.H.S. performed and analyzed experiments. Z.I.B. and M.H.S. wrote the manuscript, with feedback from all authors.

DECLARATION OF INTERESTS

The authors declare no competing interests.

Received: September 6, 2017
Revised: January 15, 2018
Accepted: March 2, 2018
Published: March 27, 2018

REFERENCES

Ahmadian, G., Ju, W., Liu, L., Wyszynski, M., Lee, S.H., Dunah, A.W., Taghibiglou, C., Wang, Y., Lu, J., Wong, T.P., et al. (2004). Tyrosine phosphorylation of GluR2 is required for insulin-stimulated AMPA receptor endocytosis and LTD. EMBO J. *23*, 1040–1050.

Anderson, W.W., and Collingridge, G.L. (2007). Capabilities of the WinLTP data acquisition program extending beyond basic LTP experimental functions. J. Neurosci. Methods *162*, 346–356.

Arroyo, S., Bennett, C., and Hestrin, S. (2014). Nicotinic modulation of cortical circuits. Front. Neural Circuits *8*, 30.

Banks, P.J., Burroughs, A.C., Barker, G.R.I., Brown, J.T., Warburton, E.C., and Bashir, Z.I. (2015). Disruption of hippocampal-prefrontal cortex activity by dopamine D2R-dependent LTD of NMDAR transmission. Proc. Natl. Acad. Sci. USA *112*, 11096–11101.

Barker, G.R.I., and Warburton, E.C. (2008). Critical role of the cholinergic system for object-in-place associative recognition memory. Learn. Mem. *16*, 8–11.

Barker, G.R.I., Bird, F., Alexander, V., and Warburton, E.C. (2007). Recognition memory for objects, place, and temporal order: a disconnection analysis of the role of the medial prefrontal cortex and perirhinal cortex. J. Neurosci. *27*, 2948–2957.

Barker, G.R.I., Banks, P.J., Scott, H., Ralph, G.S., Mitrophanous, K.A., Wong, L.F., Bashir, Z.I., Uney, J.B., and Warburton, E.C. (2017). Separate elements of episodic memory subserved by distinct hippocampal-prefrontal connections. Nat. Neurosci. *20*, 242–250.

Bennett, C., Arroyo, S., Berns, D., and Hestrin, S. (2012). Mechanisms generating dual-component nicotinic EPSCs in cortical interneurons. J. Neurosci. *32*, 17287–17296.

Brebner, K., Wong, T.P., Liu, L., Liu, Y., Campsall, P., Gray, S., Phelps, L., Phillips, A.G., and Wang, Y.T. (2005). Nucleus accumbens long-term depression and the expression of behavioral sensitization. Science *310*, 1340–1343.

Cazakoff, B.N., and Howland, J.G. (2011). AMPA receptor endocytosis in rat perirhinal cortex underlies retrieval of object memory. Learn. Mem. *18*, 688–692.

Couey, J.J., Meredith, R.M., Spijker, S., Poorthuis, R.B., Smit, A.B., Brussaard, A.B., and Mansvelder, H.D. (2007). Distributed network actions by nicotine increase the threshold for spike-timing-dependent plasticity in prefrontal cortex. Neuron *54*, 73–87.

Dani, J.A., and Bertrand, D. (2007). Nicotinic acetylcholine receptors and nicotinic cholinergic mechanisms of the central nervous system. Annu. Rev. Pharmacol. Toxicol. *47*, 699–729.

Dickerson, B.C., and Eichenbaum, H. (2010). The episodic memory system: neurocircuitry and disorders. Neuropsychopharmacology *35*, 86–104.

Evuarherhe, O., Barker, G.R.I., Savalli, G., Warburton, E.C., and Brown, M.W. (2014). Early memory formation disrupted by atypical PKC inhibitor ZIP in the medial prefrontal cortex but not hippocampus. Hippocampus *24*, 934–942.

Griffiths, S., Scott, H., Glover, C., Bienemann, A., Ghorbel, M.T., Uney, J., Brown, M.W., Warburton, E.C., and Bashir, Z.I. (2008). Expression of long-term depression underlies visual recognition memory. Neuron *58*, 186–194.

Hasselmo, M.E. (2006). The role of acetylcholine in learning and memory. Curr. Opin. Neurobiol. *16*, 710–715.

Kemp, A., and Manahan-Vaughan, D. (2007). Hippocampal long-term depression: master or minion in declarative memory processes? Trends Neurosci. *30*, 111–118.

Levin, E.D., McClernon, F.J., and Rezvani, A.H. (2006). Nicotinic effects on cognitive function: behavioral characterization, pharmacological specification, and anatomic localization. Psychopharmacology (Berl.) *184*, 523–539.

Mansvelder, H.D., and McGehee, D.S. (2000). Long-term potentiation of excitatory inputs to brain reward areas by nicotine. Neuron *27*, 349–357.

Marlin, J.J., and Carter, A.G. (2014). GABA-A receptor inhibition of local calcium signaling in spines and dendrites. J. Neurosci. *34*, 15898–15911.

Martin, S.J., Grimwood, P.D., and Morris, R.G.M. (2000). Synaptic plasticity and memory: an evaluation of the hypothesis. Annu. Rev. Neurosci. *23*, 649–711.

Papouin, T., Dunphy, J.M., Tolman, M., Dineley, K.T., and Haydon, P.G. (2017). Septal cholinergic neuromodulation tunes the astrocyte-dependent gating of hippocampal NMDA receptors to wakefulness. Neuron *94*, 840–854.e7.

Parent, M.A., Wang, L., Su, J., Netoff, T., and Yuan, L.L. (2010). Identification of the hippocampal input to medial prefrontal cortex in vitro. Cereb. Cortex *20*, 393–403.

Parikh, V., Kozak, R., Martinez, V., and Sarter, M. (2007). Prefrontal acetylcholine release controls cue detection on multiple timescales. Neuron *56*, 141–154.

Paxinos, G., and Watson, C. (1998). The Rat Brain: In Stereotaxic Coordinates (San Diego: Academic Press).

Picciotto, M.R., and Zoli, M. (2002). Nicotinic receptors in aging and dementia. J. Neurobiol. *53*, 641–655.

Picciotto, M.R., Caldarone, B.J., Brunzell, D.H., Zachariou, V., Stevens, T.R., and King, S.L. (2001). Neuronal nicotinic acetylcholine receptor subunit knockout mice: physiological and behavioral phenotypes and possible clinical implications. Pharmacol. Ther. *92*, 89–108.

Poorthuis, R.B., Bloem, B., Schak, B., Wester, J., de Kock, C.P.J., and Mansvelder, H.D. (2013). Layer-specific modulation of the prefrontal cortex by nicotinic acetylcholine receptors. Cereb. Cortex *23*, 148–161.

Ren, S.Q., Yan, J.Z., Zhang, X.Y., Bu, Y.F., Pan, W.W., Yao, W., Tian, T., and Lu, W. (2013). PKCλ is critical in AMPA receptor phosphorylation and synaptic incorporation during LTP. EMBO J. *32*, 1365–1380.

Sato, H., Kawano, T., Yin, D.X., Kato, T., and Toyoda, H. (2017). Nicotinic activity depresses synaptic potentiation in layer V pyramidal neurons of mouse insular cortex. Neuroscience *358*, 13–27.

Serrano, P., Yao, Y., and Sacktor, T.C. (2005). Persistent phosphorylation by protein kinase Mzeta maintains late-phase long-term potentiation. J. Neurosci. *25*, 1979–1984.

Sjöström, P.J., and Nelson, S.B. (2002). Spike timing, calcium signals and synaptic plasticity. Curr. Opin. Neurobiol. *12*, 305–314.

Udakis, M., Wright, V.L., Wonnacott, S., and Bailey, C.P. (2016). Integration of inhibitory and excitatory effects of α7 nicotinic acetylcholine receptor activation in the prelimbic cortex regulates network activity and plasticity. Neuropharmacology *105*, 618–629.

Verhoog, M.B., Obermayer, J., Kortleven, C.A., Wilbers, R., Wester, J., Baayen, J.C., De Kock, C.P.J., Meredith, R.M., and Mansvelder, H.D. (2016). Layer-specific cholinergic control of human and mouse cortical synaptic plasticity. Nat. Commun. *7*, 12826.

Wallace, T.L., and Bertrand, D. (2013). Importance of the nicotinic acetylcholine receptor system in the prefrontal cortex. Biochem. Pharmacol. *85*, 1713–1720.

Whitlock, J.R., Heynen, A.J., Shuler, M.G., and Bear, M.F. (2006). Learning induces long-term potentiation in the hippocampus. Science *313*, 1093–1097.

Wu-Zhang, A.X., Schramm, C.L., Nabavi, S., Malinow, R., and Newton, A.C. (2012). Cellular pharmacology of protein kinase Mζ (PKMζ) contrasts with its in vitro profile: implications for PKMζ as a mediator of memory. J. Biol. Chem. *287*, 12879–12885.

Yakel, J.L. (2014). Nicotinic ACh receptors in the hippocampal circuit; functional expression and role in synaptic plasticity. J. Physiol. *592*, 4147–4153.

Cell Reports, Volume *22*

Supplemental Information

Nicotinic Acetylcholine Receptors Control Encoding and Retrieval of Associative Recognition Memory through Plasticity in the Medial Prefrontal Cortex

Marie H. Sabec, Susan Wonnacott, E. Clea Warburton, and Zafar I. Bashir

Supplemental Information

Supplemental Information Inventory:

Supplemental Tables S1-S3

Table S1. (Related to Figure 1) Total object exploration in OiP with nAChR inactivation

Table S2. (Related to Figure 1) Total object exploration in OL with nAChR inactivation

Table S3. (Related to Figure 3) Total object exploration in object-in-place task with AMPA receptor trafficking disruption

Supplemental Experimental Procedures

Supplemental References

Table S1: Total object exploration in OiP with nAChR inactivation. Related to Figure 1

Total exploration of the four objects did not differ between control or drug condition at any time point as analysed by paired t-test. Data is presented as mean ± SEM.

Infusion	Condition	Sample Phase Exploration (s)	P Value	Test Phase Exploration (s)	P Value
Pre-Sample	Vehicle	43.1 ± 3.5	0.963	29.9 ± 2.1	0.374
	MLA	42.8 ± 3.3		27.2 ± 2.6	
	Vehicle	37.4 ± 5.1	0.794	30.4 ± 2.1	0.719
	α-BGT	36.3 ± 3.1		29.2 ± 2.6	
	Vehicle	52.6 ± 6.4	0.464	40.4 ± 3.3	0.864
	DHβE	48.9 ±4.3		41.2 ± 4.6	
Post-Sample	Vehicle	63.2 ± 4.6	0.227	46.1 ± 4.4	0.361
	MLA	54.9 ± 3.4		43.2 ± 3.9	
	Vehicle	50.3 ± 4.9	0.272	34.9 ± 3.7	0.599
	DHβE	55.7 ± 2.4		37.6 ± 2.7	
Pre-Test	Vehicle	63.8 ± 3.3	0.826	45.4 ± 6.7	0.648
	MLA	65.3 ± 5.4		43.0 ± 5.4	
	Vehicle	36.4 ± 3.7	0.762	23.5 ± 2.0	0.264
	α-BGT	35.0 ± 3.7		29.5 ± 6.1	
	Vehicle	56.7 ± 4.5	0.455	36.5 ± 3.2	0.913
	DHβE	52.2 ± 3.9		37.1 ± 4.1	

Table S2: Total object exploration in OL with nAChR inactivation. Related to Figure 1

Total exploration of the two objects did not differ between control or drug condition at any time point as analysed by paired t-test. Data is presented as mean ± SEM.

Infusion	Condition	Sample Phase Exploration (s)	P Value	Test Phase Exploration (s)	P Value
Pre-Sample	Vehicle	51.4 ± 4.5	0.482	40.7 ± 2.3	0.954
	MLA	47.5 ± 3.4		41.1 ± 5.2	
Post-Sample	Vehicle	51.3 ± 3.8	0.885	30.1 ± 4.0	0.315
	DHβE	50.8 ± 4.2		35.4 ± 3.0	

Table S3: Total object exploration in object-in-place task with AMPA receptor trafficking disruption. Related to Figure 3

Total exploration of the four presented objects did not differ between active and inactive peptide conditions at any time point as analysed by paired t-test. Data is presented as mean ± SEM.

Infusion	Condition	Sample Phase Exploration (s)	P Value	Test Phase Exploration (s)	P Value
Pre-Sample	Scr-ZIP	52.2 ± 3.7	0.379	41.3 ± 4.2	0.953
	ZIP	49.0 ± 3.8		41.0 ± 3.9	
	Scr TAT-GluA2$_{3\gamma}$	58.4 ± 3.7	0.308	34.8 ± 3.7	0.130
	TAT-GluA2$_{3\gamma}$	52.8 ± 5.5		40.4 ± 4.7	
Post-Sample	Scr-ZIP	53.4 ± 4.5	0.649	33.8 ± 3.8	0.386
	ZIP	56.2 ± 3.7		38.3 ± 2.6	
	Scr TAT-GluA2$_{3\gamma}$	59.2 ± 3.9	0.837	35.8 ± 3.3	0.835
	TAT-GluA2$_{3\gamma}$	58.5 ± 4.1		35.2 ± 3.3	

Supplemental Experimental Procedures

Animals

Male Lister Hooded rats (Harlan Laboratories, UK) were used in all experiments. Surgical procedures and behavioural tests were conducted on 34 adult rats (>300g). Subjects were removed from further testing if the implanted cannula became damaged or dislodged. Rats aged post-natal day 30 were used for slice electrophysiology. All animals were group housed under a 12hour light/dark cycle (light phase 18:00-06:00), with experimental procedures conducted during the dark phase of the cycle. Water and food was available ad libitum. All procedures were conducted in accordance with the UK Animals Scientific Procedures Act (1986), in addition to local rules and regulations of the University of Bristol.

Stereotaxic Surgery

Chronically indwelling guide cannula were implanted under inhaled isoflurane anaesthetic (Induction 4%, maintenance 2-3%; Merial, Harlow, UK). Following exposure of the skull, small burr holes were drilled and stainless steel guide cannula (26 gauge; Plastics One, Semat, UK) were targeted bilaterally into the mPFC (From bregma, AP +3.20mm; ML ±0.75mm; DV -3.5mm). Implanted cannula were secured to the skull by three stainless steel screws (Plastics One, Semat, UK) and dental cement. Dummy cannulae were inserted into guide cannula and dust caps fitted to minimise risk of post-surgical infection. Saline (5ml subcutaneous) and analgesic solution (0.05ml intramuscular; Vetergesic 0.3mg/ml) were administered for hydration and pain management on completion of the surgery. Rats were single housed for 7 days following surgery and allowed 2 weeks recovery before behavioural testing.

Behavioural Procedures

Associative recognition memory was assessed using an object-in-place (OiP) task and spatial discrimination by object-location (OL) tasks (**Fig 1A,D**). Tests were conducted in an open field arena (length; 100cm, width; 90cm, height; 50cm) with global spatial cues available. A video recorder was positioned above the arena to enable recording of exploration data. All rats were habituated to handling and the arena across 5 consecutive daily sessions.

The OiP and OL tasks consist of a sample and test phase, separated by 24h retention delay. In the OiP sample phase, rats were individually positioned in the centre of the arena and permitted free exploration of four different objects for 5mins. The objects (> 9 × 8 × 7 cm) were constructed from duplo® and were presented in the corners of the arena, 15cm from the arena walls. In the test phase (3min) two of the objects were switched in position to introduce novelty in the configuration. In the OL sample phase the animals were presented with two identical duplo® objects for 3min. Following the retention delay, the animal was replaced in the arena for the test phase, in which one object was moved into a novel position within the arena.

Exploration of each object was measured in both the sample and test phases. Exploration was counted when a rat was positioned with its nose directed towards and <2cm from an object. A minimum criterion of 15secs of total exploration during the sample phase and 10secs during the test phase was required for inclusion in statistical analysis. Exploration was scored by an experimenter blind to the drug-condition and the configuration of objects presented and moved (left or right pairs) counterbalanced within each experiment.

Novelty preference is reflected in a positive discrimination ratio (DR), calculated as the difference in the time spent exploring the moved vs unmoved objects divided by the total amount of exploration. DR=0 signifies equal exploration of moved and unmoved objects, and thus chance.

Infusions
Infusions of drug or vehicle were given to a volume of 1µL per PFC hemisphere through implanted guide cannula. Infusions delivered 15mins prior to sample phase to study effects on memory acquisition, immediately after sample exploration to study effects on memory consolidation, or 15mins before the test phase to study effects on memory retrieval. In line with previous work, infusions of TAT conjugated peptides were delivered 1hour before sample or test phases (Cazakoff & Howland., 2011). A within-subjects experimental design was used with the order of treatment (i.e. drug or vehicle) counter-balanced within experiment.

Acute Slice Preparation
Rats were decapitated under isoflurane anaesthesia and the brains were rapidly transferred into ice cold cutting solution (189mM sucrose, 10mM D-glucose, 26mM NaHCO3, 3mM KCl, 5mM MgSO47H2O, 0.1mM CaCl2, 1.25mM NaH2PO4) equilibrated with carbogen (95% oxygen, 5% carbon dioxide).

Prefrontal sections (300µm) were cut using a vibratome (Campden instruments, Ltd. Model 7000 smz2). Coronal sections were cut with a 10° angle to preserve the hippocampal afferents to the mPFC, as previously described (Parent et al., 2010). Slices were incubated at room temperature in carbogenated artificial cerebrospinal fluid (aCSF; 124mM NaCl, 3mM KCl, 26mM NaHCO3, 1.25mM NaH2PO4, 2mM CaCl2, 1mM MgSO4 and 10mM D-glucose) for at least 60mins. Post-incubation, individual slices were transferred into a recording chamber and perfused with aCSF at a rate of 2ml/min at 32.0±1.0°C (Campden instruments, Ltd. Model 7800).

Electrophysiology
Prelimbic layer V pyramidal neurons were visually identified by differential interference contrast (DIC) immersion microscopy (Olympus, LUMPlanFL N, 40x lens). Data were collected using an Axopatch 200B amplifier (Axon instruments, Ltd.) and WinLTP v1.10 software (Anderson & Collingridge., 2007), then analysed off-line. Recordings were low-pass filtered at 5kHz and digitized at 20kHz. Borosilicate glass capillaries were pulled into low resistance recording electrodes (2.5-5.5MΩ) (Sutter instruments co. Model P-87) and backfilled with intracellular solution (145mM K-gluconate, 5mM NaCl, 10mM HEPES, 0.2mM EGTA, 0.3mM GTP-Na, 4mM ATP Mg; pH 7.20-7.30, 280-290mOsM). For experiments in which peptides were loaded into the cell via the recording electrode, peptidase inhibitors (0.1mM Bestatin hydrochloride, 0.1mM Leupeptin hydrochloride) were added to the intracellular solution. Experiments were conducted in whole-cell configuration voltage-clamped to -70mV, not adjusted for liquid junction potential. Cells with series resistance values of >25MΩ, or variation >30% from baseline were discarded from analysis.

Excitatory postsynaptic currents (EPSCs) were evoked through extracellular stimulation of the hippocampal fibre bundle at 0.1Hz by a concentric bipolar electrode. Plasticity at the hippocampal-mPFC input was investigated using a paradigm of spike timing-dependent plasticity induction. A stable 5min baseline was obtained within 10mins of attaining whole-cell configuration to reduce dialysis. Cells were then held in current-clamp configuration and 80 trains of pre-post pairings were delivered at theta (5Hz) frequency. Each train consisted of 5 EPSPs at 100Hz, paired with a 7.5ms delay from the 3rd EPSP to 3 postsynaptic action potentials (APs) evoked by suprathreshold current injections (Parent et al., 2010).

Drugs
Compounds infused *in vivo* were dissolved in sterile 0.9% saline solution. Drugs used *in vitro* were dissolved in aCSF and bath applied or added to the intracellular solution for postsynaptic loading. Nicotine ditartrate, PNU-282987, RJR-2403 Oxalate, and Methyllycaconitine (MLA) were purchased

from Abcam (Cambridge Science Park, UK). Dihydro-β-erythroidine hydrobromide (DHβE), Zeta inhibitory peptide (ZIP; SIYRRGARRWRKL) and scrambled ZIP (RLYRKRIWRSAGR) were purchased from Tocris (Bristol, UK). The peptides TAT-GluR$_{3γ}$ (YGRKKRRQRRRYKEGYNVTG), scrambled TAT-GluR2$_{3γ}$ (YGRKKRRQRRRVYKYGGYNE), GluR2$_{3γ}$ (YKEGYNVYG) and GluR2$_{3A}$ (AKEGANVAG) were purchased from Eurogentec (LIEGE Science Park, Belgium. *In vivo* GluR2$_{3γ}$ and its scrambled peptide control were delivered attached to the transduction domain of the TAT protein to enable cell permeability (Brebner et al., 2005). GluR2$_{3A}$ was used for postsynaptic infusion as scrambled GluR2$_{3γ}$ was not commercially available without a conjugated TAT domain.

Statistical Analysis

Discrimination ratios (DR) were used to analyse performance in all behavioural experiments. The mean DR for each condition were compared using one-sample two-tailed t-tests against zero (chance) discrimination. Paired two-tailed t-tests were used to compare DRs between vehicle and drug conditions in individual animals. Paired t-tests were also used to compare the total time spent exploring objects in both sample and test phases of the tasks between vehicle and drug conditions.

For *in vitro* electrophysiology, the mean peak amplitude of responses recorded in the last 5mins of the baseline were compared against that of the final 5mins of plasticity by paired two-tail t-tests or repeated-measures ANOVA with Bonferroni corrected *post-hoc* comparisons. Unpaired two-tailed t-tests were used to compare the normalised response amplitudes following post-synaptic loading of active and inactive peptide. Except as stated, statistical analysis was conducted using the raw data collected, graphs are presented as mean values (±SEM) normalised to the baseline. All statistical analysis was performed using IBM SPSS 21 software package, with statistical significance was taken as $P<0.05$.

Supplemental References

Anderson, W.W., and Collingridge, G.L. (2007) Capabilities of the WinLTP data acquisition program extending beyond basic LTP experimental functions. J Neurosci Methods *162*, 346-356.

Brebner, K., Wong, T.P., Liu, L.D., Liu, Y.T., Campsall, P., Gray, S., Phelps, L., Phillips, A.G., and Wang, Y.T. (2005). Nucleus accumbens long-term depression and the expression of behavioral sensitization. Science *310* 1340-1343.

Cazakoff, B.N., and Howland, J.G. (2011) AMPA receptor endocytosis in rat perirhinal cortex underlies retrieval of object memory. Learning & Memory *18*, 688-692.

Parent, M.A., Wang, L., Su, J.J., Netoff, T. & Yuan, L.L. (2010) Identification of the hippocampal input to medial prefrontal cortex in vitro. Cereb Cortex *20*, 393-403.
